# Pentatricopeptide Repeat Gene-Mediated Mitochondrial RNA Editing Impacts on Rice Drought Tolerance

**DOI:** 10.3389/fpls.2022.926285

**Published:** 2022-07-19

**Authors:** Zhi Luo, Jie Xiong, Hui Xia, Lei Wang, Guihua Hou, Zhaoyang Li, Jing Li, Hengling Zhou, Tianfei Li, Lijun Luo

**Affiliations:** ^1^College of Plant Sciences & Technology, Huazhong Agricultural University, Wuhan, China; ^2^Shanghai Collaborative Innovation Center of Agri-Seeds (SCCAS), Shanghai Agrobiological Gene Center, Shanghai, China; ^3^Key Laboratory of Grain Crop Genetic Resources Evaluation and Utilization, Ministry of Agriculture and Rural Affairs, Shanghai, China

**Keywords:** *Oryza sativa*, pentatricopeptide repeats protein, mitochondria, RNA editing, drought, upland rice, breeding

## Abstract

Mitochondrial RNA editing plays crucial roles in the plant development and environmental adaptation. Pentatricopeptide repeat (PPR) genes, which are involved in the regulating mitochondrial RNA editing, are potential gene resources in the improvement of rice drought tolerance. In this study, we investigated genome-wide mitochondrial RNA editing in response to drought between upland and lowland rice. Responses of mitochondrial RNA editing to drought exhibit site-specific and genotype-specific patterns. We detected 22 and 57 ecotype-differentiated editing sites under well-watered and drought-treated conditions, respectively. Interestingly, the RNA editing efficiency was positively correlated with many agronomic traits, while it was negatively correlated with drought tolerance. We further selected two mitochondrial-localized PPR proteins, PPR035 and PPR406, to validate their functions in drought tolerance. *PPR035* regulated RNA editing at *rps4*-926 and *orfX*-406, while *PPR406* regulated RNA editing at *orfX*-355. The defectiveness in RNA editing at these sites had no apparent penalties in rice respiration and vegetative growth. Meanwhile, the knockout mutants of *ppr035* and *ppr406* show enhanced drought- and salt tolerance. *PPR035* and *PPR406* were under the balancing selection in upland rice and highly differentiated between upland and lowland rice ecotypes. The upland-dominant haplotypes of *PPR035* and *PPR406* shall contribute to the better drought tolerance in upland rice. They have great prospective in the improvement of rice drought tolerance.

## Introduction

RNA editing, which is widely observed in plant mitochondria and plastids, is a post-transcriptional mechanism that alters the nucleotide sequence of an RNA molecule after transcription (Hammani and Giegé, 2014). Plant RNA editing usually occurs as C-to-U conversions at the first or second position of an amino acid codon (Fujii and Small, 2011). It is hypothesized to play a role in plant adaptation to hostile environments (e.g., temperature extremes, ultraviolet radiation, and oxidative stress) when they colonize from sea to land (Fujii and Small, 2011). RNA editing changes the identity of an amino acid encoded by an organellar gene and affects its normal function (Ichinose and Sugita, 2017; Small et al., 2019). Abnormal RNA editing can result in developmental defects and alterations in stress sensitivity (Plessis et al., 2011; Sechet et al., 2015; Emami and Kempkenm, 2019).

Organellar RNA editing requires an editosome, in which the pentatricopeptide repeat (PPR) family protein is the central molecule (Hammani and Giegé, 2014; Ichinose and Sugita, 2017; Yan et al., 2018). The key structural characteristic of the PPR protein is a tandem array of ~35 amino acid repeat motifs called P-/PLS-type motif. Some PPR proteins contain the extensions of the PLS motif named as E domain and DYW domain. These conserved motifs or domains have been claimed to functions in recognizing and binding to the target sequence (P-/PLS-type motif), interacting with other proteins (E domain), and acting as cytidine deaminase (the DYW domain; Nakamura and Sugita, 2008; Yan et al., 2018; Ruwe et al., 2019). Mutations in these conserved domains could lead to dysfunction of the PPR protein and consequent defects in organellar RNA editing (Zehrmann et al., 2011; Yan et al., 2018).

Mitochondria are essential organelles in plants, in which many basic life activities, such as ATP generation *via* respiration (Schertl and Braun, 2014), ROS metabolism (Møller, 2001), and molecular signaling (Rhoads and Subbaiah, 2007; Huang et al., 2016), take place. Mitochondrial dysfunction can cause retrograde signals into the nucleus and further regulates gene expressions to adjust plant development and environmental adaptation (Gleason et al., 2011). Many loss-of-function mutants of PPR genes, which cause defects in the RNA editing at mitochondrial genes in the respiratory electron transport chain (mETC), have deleterious effects on plant growth, development, and reproduction (Kim et al., 2009; Toda et al., 2012; Liu et al., 2013; Xiao et al., 2018; Li et al., 2019; Zhang et al., 2020). Some of these PPR genes also affect plant responses to abiotic stresses or ABA, including *MEF11* (Plessis et al., 2011; Sechet et al., 2015), *AHG11* (Murayama et al., 2012), *SLG1* (Yuan and Liu, 2012), *SLO2* (Zhu et al., 2014), *POCO1* (Emami and Kempkenm, 2019), and *PPS1* (Xiao et al., 2021). It is noteworthy that the stress tolerance and growth retardation mediated by mitochondrial RNA editing occur simultaneously. For example, the loss-function mutants *slg1* (Yuan and Liu, 2012) and *slo2* (Zhu et al., 2014) possess enhanced stress tolerances while represent growth and developmental retardation due to the dysfunction of mETC. These results indicate a potential tradeoff between plant growth and stress tolerance *via* PPR-mediated mitochondrial RNA editing. This should be overcome when utilizing PPR genes in breeding.

Rice is one of the most important cereal crops. Elite paddy rice consumes a large amount of fresh water and is sensitive to drought (Luo, 2010; Luo et al., 2019). Given increasing water shortages and frequent droughts, improvement in drought resistance has been becoming a primary breeding objective of rice (Bernier et al., 2008; Luo, 2010), which requires the effective utilization of drought-resistant genes. Several studies have reported that the PPR gene and its mediated mitochondrial RNA editing are associated with rice adaptation to environmental stresses (Xiong et al., 2017; Chen et al., 2018; Xiao et al., 2021). Given the large family of PPR genes across the rice genome (Chen et al., 2018), they could be a promising gene resource for drought resistance. However, there are still no reports about the association of PPR genes and their mediated mitochondrial RNA editing with drought tolerance in rice.

Upland rice, domesticated in the drought-prone upland agroecosystem, has been adaptively differentiated from lowland rice in drought resistance at morphologic [International Rice Research Institute (IRRI), 1975; Xia et al., 2019], genetic (Lyu et al., 2014; Xia et al., 2014, 2019), transcriptomics (Luo et al., 2020), and epigenetic (Xia et al., 2016, 2017) levels. Upland rice possesses abundant genetic resources for drought resistance (Lyu et al., 2013; Zhao et al., 2018; Xia et al., 2019) and is thus widely used as the donor for drought resistance in breeding (Luo, 2010; Luo et al., 2019). Given its potential roles in rice environmental adaptation, the mitochondrial RNA editing may differ between the two rice ecotypes. Moreover, upland rice may have some beneficial variants of PPR genes in rice drought resistance.

In this study, we investigated the genetic variation of rice PPR genes, their expression responses to abiotic stresses and phytohormones, and the genome-wide mitochondrial RNA editing in upland and lowland landraces. We also functionally characterized two PPR genes, which are highly differentiated between upland and lowland rice, to elucidate their roles in rice mitochondrial RNA editing and drought resistance.

## Materials and Methods

### Genome-Wide Investigation of PPR Genes in Responses to Diverse Abiotic Stresses and Phytohormones by RNA-seq

The expression response of a gene to an abiotic stress or a phytohormone can indicate its role in the environmental adaptation and/or growth. It is therefore we have investigated the expression responses of rice *PPR* genes to four abiotic stresses and five phytohormones using Nipponbare. Rice plants were cultured in the nutrient solution proposed by Yoshida et al. (1976) in a growth chamber (16 h light at 30°C and 8 h dark at 25°C) for 15 days after germination. The 15-day-old seedlings were then treated with four abiotic stressors (15% Polyethylene glycol 6,000 (PEG6000), 1% H_2_O_2_, and 150 mm NaCl for 12 h; 10 μm antimycin A (AA) for 24 h) and five phytohormones (100 μm abscisic acid (ABA) for 12 h; 50 μm gibberellin (GA4), 50 μm auxin (IAA), 50 μm cytokinin (CTK), and 100 μm ethephon (ETH) for 4 h). Two or three biological replicates were used (Supplementary Table S1). The harvested shoot samples were sent to RNA-seq at Shanghai Majorbio Biopharm Technology Co., Ltd. and Shanghai Biozeron Biotech. Co., Ltd., using an Illumina X Ten platform (2 × 151-bp read length). The raw paired-end reads were quality controlled by Trimmomatic with default parameters.^1^ Clean reads were then separately aligned to the reference genome in orientation mode using hisat2.^2^ We then used htseq to count each gene read.^3^ The R statistical package edgeR^4^ was used to identify DEGs between the control and treatment groups using the following criteria: (1) log2 (fold change) >1 or < −1 and (2) value of *p* < 0.05. The raw data were deposited in the NCBI Sequence Read Archive (SRA; PRJNA609211).

### Genome-Wide Investigation of Genetic Diversity and Divergence Between Upland and Lowland Rice Landraces for PPR Genes

We selected 64 Geng (*japonica*) upland, 60 Geng (*japonica*) lowland landraces, and 45 wild rice accessions (Supplementary Table S2) from China to investigate the genetic diversity and divergence of PPR genes among them. These plant materials were previously resequenced and the SNPs of each PPR gene (−2000 bp to +2000 bp) were extracted (Luo et al., 2020). Genetic diversity (π) was calculated for all genes in upland rice, lowland rice, and wild rice based on their SNPs. The relative π ratios (π_upland_/π_lowland_ and π_landrace_/π_wild_) of the PPR genes were calculated and compared with the genomic average. Meanwhile, the F_ST_ of each PPR gene between upland and lowland (U-L F_ST_) or between landrace and wild rice (C-W F_ST_) was also calculated and compared with the genomic average by the single-sample Kolmogorov–Smirnov test. If a PPR gene is highly differentiated between upland and lowland rice, it could be a candidate for rice drought adaptation.

### Genome-Wide Investigation of Mitochondrial RNA Editing Among Diverse Rice Genotypes

#### Field Experiment to Evaluate Agronomic and Drought-Resistant Traits

The above-mentioned 64 upland and 60 lowland landraces from China were used to evaluate their agronomic and drought-resistant traits in the drought resistance screening facility at the Baihe Experimental Station, Shanghai, in 2016 (May to October). The procedure of the field experiment was described in detail in a previous study (Luo et al., 2020). Briefly, plants of each rice genotype were planted in two nearby fields: one was treated with a drought (the DT field) from July 5th to August 15th, while the other remained well-watered by drip irrigation and served as the control (the CK field). Rice seedlings were transplanted into plots with 8 rows × 8 hills at 18-cm intervals on June 15 and 30 days after germination. Ten agronomic traits, namely, plant height (PH), number of tillers (NT), flag leaf length (FLL), flag leaf width (FLW), number of grains per plant (NG), 100-grain weight (100GW), grain yield per plant (GY), aboveground biomass, harvest index (HI), and fecundity were measured in the DT and CK fields. These traits were measured from six individuals. The relative water content (RWC) was measured from three replicates on August 5th in the CK and DT fields. Most of the rice plants were at booting stage and exhibited significant leaf-rolling phenomenon in the DT field. Most of the rice plants were at booting stage and exhibited significant leaf-rolling phenomenon in the DT field. It was calculated as follows: (weight of fresh leaf - weight of dry leaf)/ (weight of saturated leaf - weight of dry leaf).

#### Evaluation of Mitochondrial RNA Editing Among Rice Landraces, Modern Cultivars, and Wild Rice Accessions

For rice landraces, three top leaves from three individuals for each genotype were mixed and harvested on the same day when RWC was measured (August 5th) in the CK and DT fields, respectively. The leaf samples were flash-frozen and stored in liquid nitrogen until RNA extraction. For modern cultivars and wild rice accessions (Supplementary Table S2), plants of each genotype were planted into a plot of 8 rows × 8 hills with 18-cm intervals in the well-managed paddy field at the Baihe Experimental Station, Shanghai, in 2017. The leaf samples were harvested on August 20^th^ with three replicates for each genotype.

Total RNA was extracted using the TRNzol-A+ Total RNA Reagent (TIANGEN, Beijing, China). cDNA for each sample was obtained from reverse transcription of total RNA using the PrimeScript RT reagent Kit (Takara Biotechnology, Dalian, China) according to the manufacturer’s instructions. Sixty target regions from thirty-one mitochondrial genes (Supplementary Table S3) were selected for RNA editing by the FastTarget™ technology at Genesky Biotechnologies Inc., Shanghai. In brief, the target regions were amplified by multiplex PCR using their corresponding primers (Supplementary Table S4). A unique index tag was added to the PCR products for each cDNA sample. PCR products with sample-specific tags were mixed equally and used to construct the library. The library of mixed samples was sequenced using the Illumina NextSeq500 platform. Quality-controlled reads were mapped to the reference mitochondrial gene sequences. The number of SNP counts (C to T or G to A) was analyzed based on the total mapped reads. The editing efficiency at one site was calculated as: counts of altered bases (T or A)/total counts of bases (C + T or G + A). We defined the informative editing site when it meets three sceneries below: (1) the editing site should be from C to T or G to A; (2) number of genotypes that is fully edited (100%) or non-edited (0%) at this site is less than 95%; and (3) the missing data is less than 10%. To ensure the reliability of this method, we selected three mitochondrial genes to validate the editing efficiency. Target regions of these selected mitochondrial genes were amplified using the corresponding primers from cDNA (Supplementary Table S4). The amplified band of each mitochondrial gene was recycled from the PCR product on an agarose gel and then transformed into *Escherichia coli*. Thirty clones were selected and sequenced by the Sanger method to count the ratio of C to T conversions. A good correlation was observed between the editing efficiency estimated by the high-throughput sequencing and that estimated by the Sanger method (Supplementary Figure S1).

First, the drought-responsive site was defined as its editing efficiency exhibiting a significant difference between CK and DT conditions by paired *t*-test. Second, the ecotype-differentiated site was defined when a significant difference was detected in its editing efficiency between rice ecotypes by independent *t*-test. Finally, three response patterns of RNA editing at a site were defined: (1) the upregulated site, whose alteration ratio of RNA editing [(E_d_−E_w_)/E_w_] is larger than 0.2; (2) the downregulated site, whose alteration ratio is less than −0.2; and (3) unchanged site, whose alteration ratio is between −0.2 and 0.2. Here, E_d_ indicates editing efficiency in DT, and E_w_ indicates editing efficiency in CK. We also compared the RNA editing efficiency between modern cultivars and wild rice accessions by independent *t*-test.

#### Correlation Analyses

First, we applied the correlation analysis (Pearson correlation coefficient, PCC) among all sites based on their editing efficiencies measured from the CK and DT fields, respectively. Second, we applied the correlation analysis between the editing efficiency at each site and each measured trait among rice landraces. The significance of the correlation was determined by the following criteria: |PCC| > 0.2 and *p* < 0.05. The correlation of RNA editing at a mitochondrial gene with a trait was determined by the largest PCC value among all sites of the mitochondrial gene. However, we should point out that the mitochondrial RNA editing is dynamic which means the correlations between RNA editing at the booting stage and the morphological traits measured after harvest (e.g., PH, NT, NG, GY, etc.) are for reference only.

## Functional Characterization of *PPR035* and *PPR406*

### Plant Materials

Two mitochondrial-localized *PPR* genes (*PPR035* LOC_Os01g46230 and *PPR406* LOC_Os10g30760) were selected for functional characterization. The modified CRISPR-Cas9 method (Ma et al., 2015) was used to obtain knockout mutants for the candidate PPR genes in Nipponbare (wild type, WT). The primers used for CRISPR-Cas9 are listed in Supplementary Table S4. Three independent knockout mutants of each gene (coded as *ppr035*-1,2,3 and *ppr406*-1,2,3) were identified from T0 transformants by Sanger sequencing at the designed target region (Supplementary Figures S2a,b). They were bred to generate T3 for further field and laboratory experiments.

### Comparison of Mitochondrial RNA Editing Between Knockout Mutants and WT Plants

We tested the RNA mitochondrial editing efficiencies in the transgenic and WT plants from 15-day-old seedlings by the above-described method. Three replicates were designed for each rice material. The independent *t*-test was applied to detect any significant differences in the editing efficiency at a site between the mutants and WT.

### Subcellular Localization

The full-length *PPR035* and *PPR406* without the stop codon were cloned into the pan580-GFP destination vector using Gateway cloning, and the primers are described in table MM3. The protoplasts were extracted from 10-day-old etiolated rice seedlings and then transformed with 1.5–10 μg plasmids. To confirm mitochondrial localization, the mitochondrial-localized protein mstp fused to RFP (Heazlewood et al., 2004) was used as a mitochondrial-specific marker. The organelle and GFP signals were detected with a confocal microscope (Nikon, C2-ER, Tokyo, Japan) at different excitation wavelengths.

### Evaluation of the Growth and Reproduction Traits for *ppr035* and *ppr406*

The respiration ability of WT, *ppr035*, and *ppr406* was quantified by the oxygen consumption rate measured with Oxytherm system (Hansatech, Kings Lynn, United Kingdom). Briefly, 0.3 g well-chopped fresh leaf samples from 15-days-old seedlings were injected into the reaction chamber with 2 ml air-saturated K_2_HPO4 buffer (20 mm and pH = 6.0). The reaction temperature was set at 25°C, and the speed of the magnetic stirrer was set to 100. The oxygen depletion curve was generated to estimate oxygen consumption rates from a 4-min duration after its slope became stable. Four biological replicates were used for the measurements.

We then tested the germination behavior and early growth of WT, *ppr035*, and *ppr406*. A culture dish (8 cm diameter) containing 30 well-matured seeds of plant material was designed as a replicate, and three replicates were involved. The seeds were germinated in a growth chamber (25°C without light) with 12 ml fresh water, 12 ml 20% PEG6000 solution, and 12 ml 180 mm NaCl solution, respectively. To estimate the early growth, at least 10 young seedlings of each material were selected to measure their root lengths and shoot dry weights at 5 days after germination in a growth chamber (16 h light at 30°C and 8 h dark at 25°C).

Field experiments were conducted in regularly managed paddy fields at Linshui Experimental Station, Hainan (2019.12–2020.5). Rice seedlings were transplanted into a plot with 4 rows × 8 hills with 20 cm intervals with three replicates (plots) following a randomized complete block design. PH, NT, biomass, 100GW, fecundity, GY, and HI were measured from at least eight plants in each plot.

### Evaluation of Drought Avoidance, Drought Tolerance, and Salt Tolerance in *ppr035* and *ppr406*

We evaluated the root gravitropism and excised leaf water loss rate (EWR) to estimate drought avoidance. The root gravitropism was quantified using the modified method described in Uga et al. (2013). Briefly, six germinated seeds (embryos downward) were sowed evenly onto a plate (12 × 10 × 1.2 cm) filled with 0.8% agarose gel. The plate was placed in an incubator at 28°C in the dark. Approximately 1 day later, when the radicle root grew to a length of 1 to 2 cm, a tangent line to the growth direction was marked on the board. The plate was then rotated 90° and immediately placed back into the incubator. One hour later, a new tangent line to the growth direction of the new root tip was marked. The bending angle between the two tangent lines was used to quantify the root gravitropism. At least 10 individuals were measured for each plant material. The EWR at a time (T_n_) was calculated as follows: (*W*_T0_−*W*_Tn_)/(*W*_T0_−*W*_dry_). *W*_T0_ is the fresh weight of the rice shoot when it was excised (T0). *W*_Tn_ is the weight of the shoots at T_n_. *W*_dry_ is the weight of the completely dried shoots. The EWR was measured in six 15-day-old seedlings for each plant material.

For drought and salt tolerance, 48 rice seedlings of each plant material were grown in a 96-well plate (as a replicate) in the nutrient solution (Yoshida et al., 1976) for 15 days in the growth chamber. Three replicates were used for each experiment. The 15-day-old seedlings were then treated with 20% PEG6000 and 180 mm NaCl for 5 days before recovery. Seven physiological traits, including RWC, total soluble protein (A045-2-2), total soluble sugar (A145-1-1), total anti-oxidant capacity (AOC, A015-1-2), proline content (A107-1-1), the specific activity of superoxide dismutase (SOD, A001-3-2), and the specific activity of catalase (CAT, A007-1-1) were measured before the treatment and 2 DAT. The H_2_O_2_ content (A064-1-1) was measured before treatment and 4 DAT. The measurements of other physiological traits were performed following the manufacturer’s instructions for their corresponding test kits from Nanjing JianCheng Bioengineering Institute, China.^5^ Three biological replicates were used, and one replicate contained four mixed harvested samples. The survival ratio was measured 3 day after recovery.

### Expression Features of *PPR035* and *PPR406* Quantified by qPCR

We quantified the spatial and temporal expression patterns of *PPR035* and *PPR406* using qPCR in the WT. We also tested the expressions of *PPR035* and *PPR406* in rice seedlings treated with osmotic (20% PEG6000), salinity (180 mm NaCl), oxidative (50 mm H_2_O_2_), ABA (10 μm), low temperature (5°C), and higher temperature (42°C) for 2, 4, and/or 8 h. As the expression of genes encoding the alternative oxidases (AOX*s*) is a molecular feature of mETC dysfunction (Li et al., 2019), expressions of *AOX1a*, *AOX1b*, and *AOX1c* was quantified in WT and mutants by qPCR. We also quantified the expression of *rps4* and *orfX* by qPCR among WT and mutant lines. The reference for nucleus-encoded and mitochondrion-encoded genes (e.g., *PPR035, PPR406, AOX1a,* etc.) in qPCR was *Actin* and *ATP6*, respectively.

### Transcriptomic Alterations in the Knockout Mutants of *PPR035* and *PPR406*

To explore the potential impacts on rice transcriptome by the knockout of *PPR035* and *PPR406*, three biological replicates of 15-day-old WT, *ppr035*-1/2, and *ppr406*-1/2 plants were sampled for RNA sequencing. The genes differentially expressed between mutants and WT were identified as DEGs in *ppr035* or *ppr406*. Enrichment analyses of Gene Ontology (GO) and Kyoto Encyclopedia of Genes and Genomes (KEGG) were applied based on the DEGs detected in *ppr035* or *ppr406*. In addition, to determine the common impacts of *PPR035* and *PPR406* on rice transcriptome, we also defined the core DEG as Type I, a gene determined as the DEG in both *PPR035* and *PPR406*; and Type II, a gene determined as the DEG in at least one mutant line, while its |log2(fold change)| > 2 in all the four mutant lines. The subcellular localization of the core DEGs was predicted using TargetP.^6^ Gene expression quantified by RNA-seq was validated by qPCR from three core DEGs (*OsHAC1; 2*, *OCPI1*, and *OsRAA1*; Supplementary Figure S3). In addition, we tested the expression responses of these core DEGs to temporal (2, 4, 12, 24 h) treatments of 100 μm ABA, 1% H_2_O_2_, 15% PEG6000, and 150 mm NaCl in Nipponbare by RNA-seq (Supplementary Table S3).

### Explore the Roles Played by *orfX* or *rps4* in Rice Drought Tolerance

*PPR035* and *PPR406* regulated RNA editing at *orfX* and/or *rps4*. To test whether the enhanced drought and salt tolerance in *ppr035* and *ppr406* were caused by their roles in RNA editing at *orfX* and/or *rps4*, we first investigated their expression responses to the osmotic stress (20% PEG6000 for 4 and 8 h), salinity stress (180 mm NaCl for 4 and 8 h), oxidative stress (1% H_2_O_2_ for 4 and 8 h), ABA (100 μm ABA for 4 and 8 h), low temperature (5°C for 2 and 4 h), and higher temperature (42°C for 2 and 4 h) by qPCR using *ATP6* as the reference. Meanwhile, we classified 20 rice landraces as high expression genotypes (HEGs) and low expression genotypes (LEGs) in terms of *orfX* and *rps4* expressions quantified by qPCR. We then tested any differences in the expression of the core DEGs between HEGs and LEGs using an independent *t*-test. Three biological replicates were involved in qPCR. The primers used in qPCR are listed in Supplementary Table S4. The expression levels of the core DEGs in landraces quantified by RNA-seq was obtained from the previous study (Luo et al., 2020).

Although the function of orfX in plant is still not clear, it contains a conserved domain that is similar to Sec-independent protein translocase protein (TatC) annotated in NCBI.^7^ The TatC is predicated to have trans-membrane helices (TMHs) and might be involved in twin-arginine signal peptide recognition, protein translocation, and proton translocation, which is related to mitochondrial biogenesis (Carrie et al., 2016). To study the potential influence of RNA editing on the function of orfX, we applied predictions of trans-membrane helices for the intact, fully edited, and defectively edited (SNV01178 and SNV01187) orfX by their corresponding amino sequences (Supplementary Figures S4a,b) *via* the sub module MEMSAT-SVM (Membrane Helix Prediction) of SECONDARY STRUCTURE PREDICTION (PSIPRED^8^ Nugent and Jones, 2009).

Prokaryotic expression system could be used for the verification of some plant stress tolerance genes (Deeba et al., 2020; Yang et al., 2020). We constructed prokaryotic expression vector pGEX-6p1-orfX to evaluate the potential functions of orfX in drought and salt tolerance. The full-length CDS of *orfX* was amplified using specific in-fusion primers (Supplementary Table S4) and inserted into the BamHI and EcoRI digested pGEX-6p-1vector (GE Healthcare). The yielded plasmid of pGEX-6p1-orfX was then transformed into *E. coli* strain BL21 (DE3, Transgene). A strain transformed with pGEX-6P-1 was used as a control. All of the transformed *E. coli* BL21 strains were grown in 5 ml cultures at 37°C for OD600 near 0.4–0.6. Then the growth in normal condition was measured by further culturing in 20 ml LB medium containing Ampicillin (100 μg/ml). For abiotic stress treatments, 0.6 M sorbitol and 0.5 M NaCl was added into the medium, respectively. To induce the expression of the target proteins, 0.2 mm IPTG (Isopropyl β-D-1-thiogalactopyranoside) was also added to the culture solutions. Initial cell concentrations were adjusted to be equivalent in each flask in these experiments. The velocity of the shaker was 200 rpm min^−1^ and the growth temperature was set at 37°C. The concentration of *E. coli* cells (OD600) at each time point was determined. Each experiment was replicated three times.

### Evolution of *PPR035* and *PPR406* in Rice

We conducted haplotype analysis among rice landraces and wild rice accessions using the SNPs (−2000 bp to +2000 bp) called from resequencing. PopART (ver. 17) was used to visualize the haplotype network. The dominant haplotypes in the typical upland and lowland rice were coded as *PPR035^U^*/*PPR406^U^* and *PPR035^L^*/*PPR406^L^*, respectively. Any differences in RNA editing efficiencies at the three sites (SNV00404, SNV01178, and SNV01187), agronomic traits, and expression levels of the core DEGs between landraces of *PPR035^U^*/*PPR406^U^* and *PPR035^L^*/*PPR406^L^* were tested by the independent *t*-test. In addition, protein structures of PPR035^U^/PPR406^U^ and PPR035^L^/PPR406^L^ were predicted using Phyre2 with default parameters (Kelley et al., 2015).

## Results

### Expression of PPR Genes in Responses to Abiotic Stresses and Phytohormones

First, we tested the genome-wide expressions of PPR genes in responses to various abiotic stresses and phytohormones. Among 491 PPR genes predicted across the rice genome, 482 of which were expressed in at least one of our sequenced samples (Supplementary Material). There were 16/81, 15/127, and 27/35 PPR genes that were upregulated/downregulated in responses to osmotic, salt, and oxidative stress, respectively (Supplementary Figure S5a). Among the phytohormones-responsive genes, 12/7, 87/21, 77/96, and 74/88 were upregulated/downregulated in responses to ethylene (ETH), gibberellin (GA), auxin (IAA), and cytokinin (CTK), respectively (Supplementary Figure S5a). A considerable proportion of PPR genes had crosstalk-responses between different abiotic stresses (24.4 ~ 37.1% between osmotic, salt, and oxidative stresses) or between different phytohormones (28.7 ~ 36.7% between GA, IAA, and CTK). Meanwhile, 4.5–20.2% PPR genes were at least in response to one abiotic stress and one phytohormone (Table 1). Noticeably, more PPR genes were downregulated under the osmotic, salinity, and oxidative stresses, while the number of upregulated and downregulated PPR genes under treatments of GA, IAA, and CTK were equivariant (Supplementary Figure S5a). Meanwhile, PPR genes of P and DYW types contained more members which were responsive to the abiotic stresses and phytohormones (Supplementary Figure S5b). A considerable proportion of mitochondrion- and chloroplast-localized PPR genes were in responses to the abiotic stresses and phytohormones (Supplementary Figure S5c). Based on the above observations, we considered that PPR genes of P and DYW types that were localized in mitochondria and chloroplasts potentially played a role in rice growth and environmental adaptation.

**Table 1:** Number (below matrix) and proportions (above matrix) of PPR genes in responses to a pair of treatments.

Treatments	Osmotic	Salt	Oxidative	ABA	ETH	GA	IAA	CTK
Osmotic	/	34.3%	37.1%	8.7%	4.5%	12.3%	16.0%	14.1%
Salt	61	/	24.4%	6.8%	5.2%	15.8%	20.2%	18.8%
Oxidative	43	40	/	11.6%	5.2%	12.7%	11.9%	7.7%
ABA	9	10	8	/	13.3%	8.0%	4.4%	2.9%
ETH	5	8	4	4	/	4.1%	4.3%	4.6%
GA	22	34	19	9	5	/	30.2%	28.7%
IAA	39	53	25	8	8	65	/	36.7%
CTK	32	48	16	5	8	60	90	/

### Evolution of PPR Genes During Rice Domestication and Ecotypic Differentiation

The relative genetic diversity and divergence of a PPR gene between upland and lowland rice ecotypes can provide valuable cues for its evolution during rice domestication. The mean relative diversity (π_Cultivar_/π_Wild_ and π_Upland_/π_Lowland_) and genetic divergence (F_ST(C-W)_ and F_ST(U-L)_) of the total PPR genes were generally equivalent to the genomic average (Supplementary Table S5). PPR genes of most categories had equivalent levels of relative diversity and genetic differentiation to the average of the total PPR genes (Supplementary Table S5). We detected 44 highly differentiated PPR genes (F_ST_ > 0.305, beyond the 95% confidence interval), including 26 (59.1%) mitochondrion-localized genes, between upland and lowland rice ecotypes (Supplementary Material 1).

### Agronomic and Drought-Resistant Traits Measured From Upland and Lowland Rice Landraces

Drought had a strong negative impact on rice growth and reproduction, representing significant decreases in PH, NT, FLW, biomass, fecundity, GY, and HI under stressed conditions (Supplementary Table S6). Significant differences in many measured traits were observed between the rice ecotypes. In the CK field, lowland rice exhibited more tillers, narrower flag leaves, a higher GY, more biomass, a higher HI, and better fecundity (Supplementary Table S6). In the DT field, upland rice exhibited a greater PH, fewer tillers, and longer and wider flag leaves (Supplementary Table S6). The leaf water status, revealed by the RWC, greatly decreased under drought conditions (Supplementary Table S6), indicating a severe degree of drought in the experiment. Upland rice possessed better drought tolerance, as indicated by the higher RWC and relative GY in the DT (Supplementary Table S6).

### Mitochondrial RNA Editing in Responses to Drought

**Figure 1:**
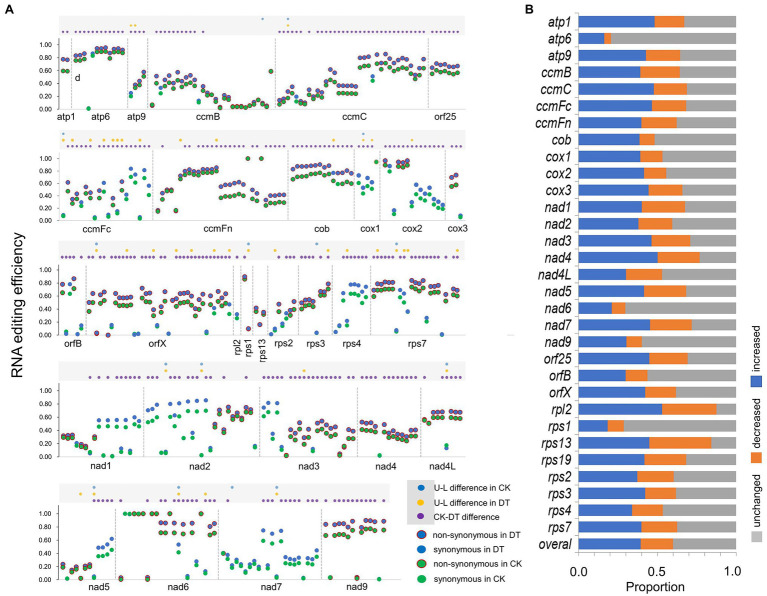
Rice mitochondrial RNA editing in well-watered (CK) and drought-treated (DT) fields. **(A)** RNA editing efficiencies of upland (U) and lowland (L) rice at 426 informative sites in well-watered (CK) and drought-treated (DT) fields. The dots within gray rectangle on top mark the significant difference in editing efficiency between rice upland (U) lowland (L) or treatments. **(B)** Proportion of increased, decreased, and unchanged editing efficiencies of genotype-site combinations at each mitochondrial gene.

We detected 426 informative editing sites with the averaged depth > 500 (Supplementary Material 2). Editing efficiency varied considerably among sites and genotypes (Figure 1A; Supplementary Table S7). It is noteworthy that the PCCs (Pearson Correlation Coefficients) of adjacent sites and sites of the same mitochondrial gene were significantly higher than those of unrelated sites (Supplementary Figure S6), indicating that RNA editing at sites within the same mitochondrial gene could be synchronously regulated.

The efficiency of RNA editing among all informative sites generally increased in response to drought (Figure 1A). Based on the paired *t*-test, 240 sites increased their editing efficiencies in response to drought, while only two sites decreased their editing efficiencies in response to drought (Figure 1A; Supplementary Table S7). However, we also observed great variations in the drought-response patterns of RNA editing among sites, genes, and genotypes (Figure 1). We can observe that the RNA editing at *atp6*, *nad6*, and *rps1* was stable with the least upregulated and downregulated site-genotype combinations, while that at was more changeable *rpl2* and *rps13* with the most upregulated and downregulated site-genotype combinations (Figure 1B).

When comparing between ecotypes by independent *t*-test, we detected 22 and 57 ecotype-differentiated sites (including 11 common sites), whose editing efficiencies exhibited significant differences between the two rice ecotypes under CK and DT conditions, respectively (Figure 1A). Meanwhile, we detected 25 ecotype-differentiated nonsynonymous sites. They were distributed mainly in mitochondrial genes related to cytochrome C (*ccmC* and *ccmFc*), small ribosomal subunit (*rps1*, *rps2*, *rps7*, and *rps13*), and *orfX* (Figure 1A).

### Correlations Between the Mitochondrial RNA Editing and the Measured Agronomic/Drought-Resistant Traits

**Figure 2:**
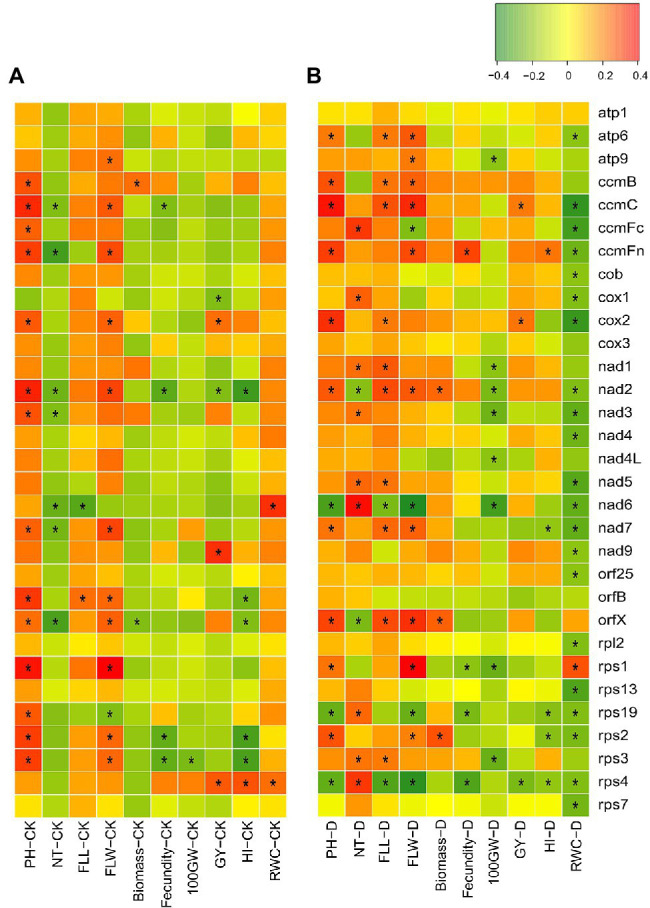
Correlation between RNA editing efficiencies of a mitochondrial gene and a measured trait in **(A)** well-watered (CK) and **(B)** drought-treated (D) fields. ^*^ indicates the significant correlation (|PCC| > 0.20 and *p* < 0.05). PH, plant height; NT, number of tillers; FLL, flag leaf length; FLW, flag leaf length, 100GW, 100-grain weight; GY, grain yield per plant; HI, harvest index; RWC, relative water content.

In CK, there were 54 positively correlated site-trait combinations and 22 negatively correlated site-trait combinations (Supplementary Table S8). At the gene scale, RNA editing at 14 and 11 mitochondrial genes was positively correlated with PH and FLW, respectively (Figure 2A). In contrast, RNA editing at 6, 4, and 5 mitochondrial genes was negatively correlated with NT, fecundity, and HI, respectively (Figure 2A). Under DT conditions, there were 104 positively correlated site-trait combinations and 175 negatively correlated site-trait combinations (Supplementary Table S8). As the editing efficiency was estimated at booting stage while the agronomic traits were measured after harvest, the information from correlations between RNA editing and the agronomic traits is limited. We paid particular attentions to the correlation between RNA editing and RWC as their samples were harvested on the same day. Interestingly, editing efficiencies of nearly one third editing sites (139 of 426), were negatively correlated with RWC under DT (Figure 2B). They were distributed in 21 mitochondrial genes (Supplementary Table S8). These RWC negatively correlated sites indicated that the lower RNA editing efficiency at these editing sites were potentially associated with the better drought adaptation.

### *PPR035* and *PPR406* Were Involved in RNA Editing at *rps4* and *orfx*

We selected two PPR genes (*PPR035* and *PPR406*) for functional validation, as they were highly differentiated between ecotypes (F_ST_ = 0.713 and 0.574, placed in first and third by F_ST_), predicted and then proven to be targeted in mitochondria (Supplementary Figure S7), and downregulated by osmotic and salinity stresses (Supplementary Figures S8a,b). *PPR035* and *PPR406* were expressed mainly in leaf tissues throughout the rice life history (Supplementary Figures S8c,d).

Using the CRISPR-Cas9 method, we created three independent stop-gain mutants for each of the two *PPR* genes (coded as *ppr035*-1-3 and *ppr406*-1-3; Supplementary Figures S1a,b). In *ppr035*, the RNA editing efficiency at two nonsynonymous editing sites, (SNV00404 for *rps4*-926 and SNV01178 for *orfX*-406), decreased to zero (Supplementary Figure S9a; Supplementary Table S9). In *ppr406* mutants, we detected defects in RNA editing at one nonsynonymous editing site at *orfX* (SNV01187 for *orfX*-355; Supplementary Figure S9a; Supplementary Table S9). Editing efficiencies at all three sites were negatively correlated with RWC-DT (Supplementary Table S8). Among these editing sites, SNV01187 is an ecotype-different site in both CK and DT. Besides, *rps4* and *orfX* also contained several other ecotype-differentiated sites (SNV00405, SNV01176, SNV01177, and SNV01179; Figure 1A), which were also negatively correlated with RWC-DT (Supplementary Table S8). However, we did not observe any significant changes in the expression levels of *rps4* and *orfX* in *ppr035* and *ppr406* compared to the wild type (WT) under the normal and osmotic treatments (Supplementary Figures S9b–j).

### *PPR035* and *PPR406* Negatively Impact on Rice Drought and Salt Tolerance by Regulating a Network of Stress-Responsive Genes

**Figure 3:**
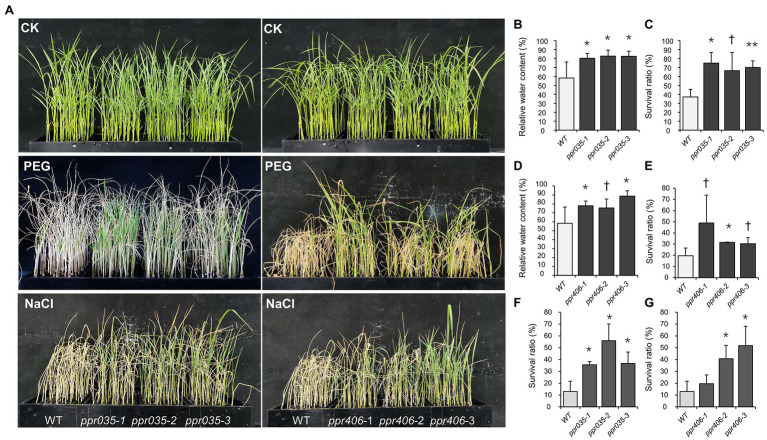
Performance of the wild type (WT) and knockout mutants (*ppr035*-1/2/3 and *ppr406*-1/2/3) under osmotic and salt stress. **(A)** Performance of plant materials in the treatments with normal nutrient solution (CK), 20% PEG6000, and180mM NaCl. **(B,C)** Relative water content and survival ratio of WT and *ppr035*-1/2/3 under osmotic stress. **(D,E)** Relative water content and survival ratio of WT and *ppr406*-1/2/3 under osmotic stress. **(F,G)** Survival ratio of WT, *ppr035*-1/2/3, and *ppr406*-1/2/3 under salt stress. ^†^, ^*^, and ^**^ indicate significances at *p* < 0.1, *p* < 0.05, and < 0.01 by independent *t*-test between WT and mutants.

**Figure 4:**
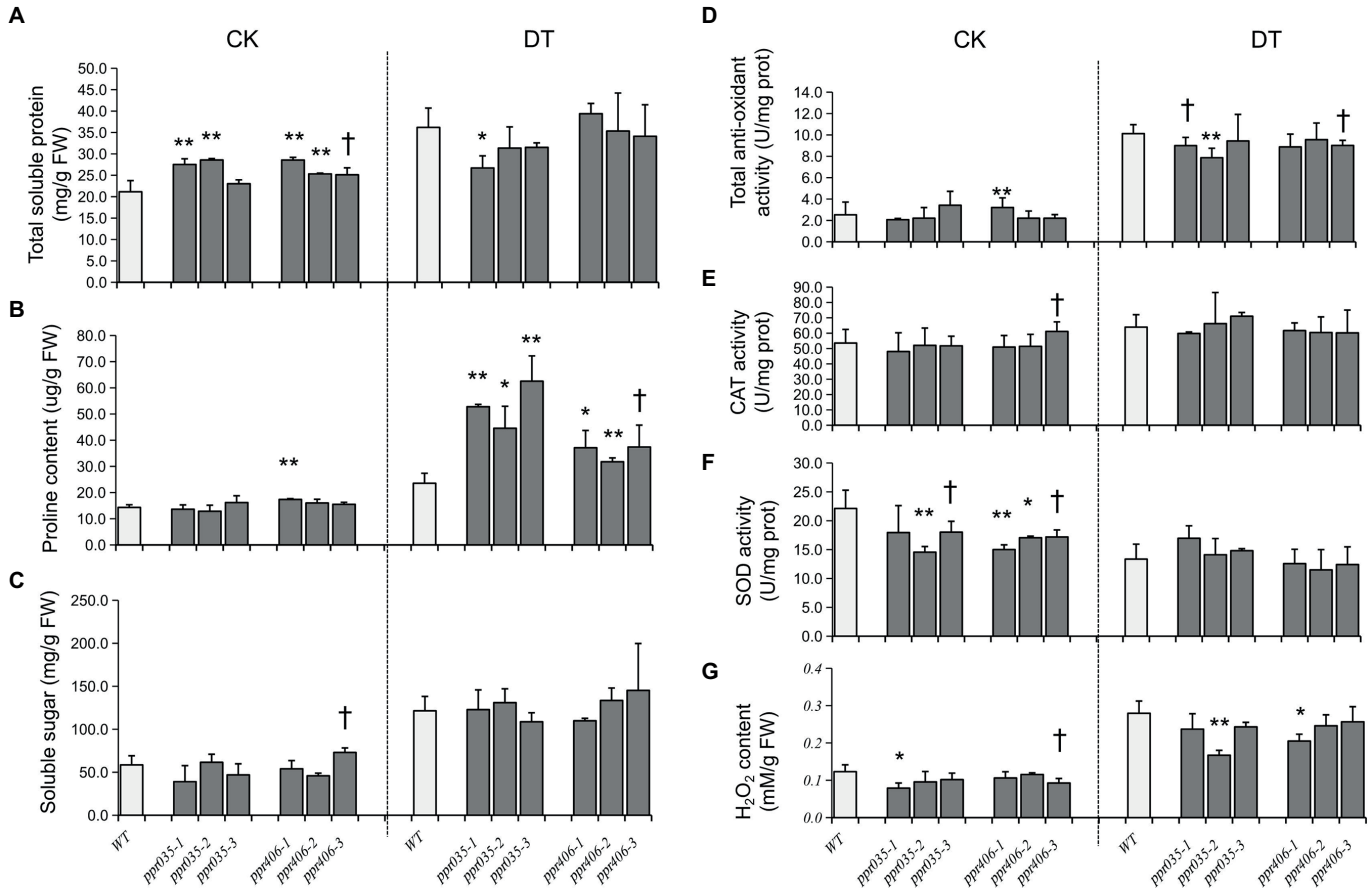
Physiological traits measured from WT, *ppr035*, and *ppr406* in normal (CK) and PEG6000 simulated osmotic stress (DT). **(A)** Total soluble protein. **(B)** Proline content. **(C)** Soluble sugar. **(D)** total anti-oxidant capacity. **(E)** SOD activity. **(F)** CAT activity. **(G)** H_2_O_2_ content. ^†^, ^*^, and ^**^ indicate significances at *p* < 0.1, *p* < 0.05, and < 0.01 by independent *t*-test between WT and mutants.

As upland and lowland rice ecotypes are differentiated in drought resistance, we evaluated drought avoidance and drought tolerance for *ppr035* and *ppr406*, respectively. At the three-leaf stage, seedlings of *ppr035* and *ppr406* exhibited no detectable difference in vegetative growth (Supplementary Figures S10a–d) but improved tolerance to the PEG-simulated osmotic stress, presented as a higher RWC at 2 days after treatment (DAT) and the better survival after recovery from 5 DAT (Figures 3A–E). The total soluble protein was significantly higher in mutants in CK, while it was similar between the WT and mutants in DT (Figure 4A). The proline content, which is associated with plant osmotic adjustment, exhibited significant differences between mutants and WT plants (Figure 4B). In contrast, phycological traits related to ROS scavenging (e.g., SOD, CAT, and AOC) were similar between mutants and WT (Figures 4D–F). Meanwhile, the H_2_O_2_ content was somewhat lower in the mutants (Figure 4G), reflecting fewer negative impacts by drought. By investigating traits of drought avoidance, *ppr035* and *ppr406* showed similar EWR (Supplementary Figures S11a,b) and root gravitropism (Supplementary Figures S11c,d) with the WT. These results indicate that *PPR035* and *PPR406* have impacts on drought tolerance rather than drought avoidance. In addition, *ppr035* and *ppr406* also possessed higher survival ratios under the salinity stress at the seedling stage (Figures 3A,F,G).

By comparing the transcriptomes of *ppr035* and *ppr406* with that of WT, we detected 2,103 differentially expressed genes (DEGs) among the four mutant lines in total (Supplementary Table S10; Supplementary Figure S12a). Based on the DEGs detected, *PPR035* and *PPR405* shared many common enriched GO terms (e.g., mitochondrion, DNA-banding transcription factor activity, response to stimulus, and signal transduction) and KEGG pathways (e.g., starch and sucrose metabolism, diterpenoid metabolism, monoterpenoid metabolism, etc.; Supplementary Figures S12c–f). We identified 125 core DEGs that were mainly located in the endoplasmic reticulum (33 genes) and mitochondrion (22 genes) predicted by TargetP (Supplementary Table S10). Among the core DEGs, we noticed that several known drought-tolerant genes related to osmotic adjustment (*OCPI1, OsLEA3-2, ZFP36,* and *CYP94C2b*) were highly upregulated in the four mutant lines (Supplementary Table S10), which may explain the improved drought tolerance in *ppr035* and *ppr406*. Most of these core DEGs were in responses to ABA, oxidative, osmotic, and/or salinity stress (Supplementary Figure S13). Therefore, we considered that *PPR035* and *PPR406* regulate drought tolerance *via* these core DEGs.

### *ppr035* and *ppr406* Have No Obvious Impact on the Vegetative Growth

Defects in mitochondrial RNA editing, particularly those at mitochondrial genes encoding proteins of the ETC complex, could always lead to abnormal mitochondria, dysfunction of respiration, and consequent growth and developmental retardation (Sosso et al., 2012; Zhu et al., 2012; Xiao et al., 2018). In this study, we did not observe apparent alterations in respiration, as revealed by the oxygen consumption rate in *ppr035* and *ppr406* mutants (Supplementary Figures S14a–d) and equivalent expressions of *AOXs* (Supplementary Figures S14e–g) in mutants to those in WT. This may be because the proteins encoded by *orfX* and *rps4* are not the vital parts of the ETC complex. In addition, the DEGs in *ppr035* and *ppr406* rarely overlapped with antimycin A-responsive genes (Supplementary Figure S12b). As a result, the knockout of *PPR035* and *PPR406* had no apparent effects on seed germination (Supplementary Figures S15a,b) or vegetative growth (Supplementary Figures S15c–f, S16a–c,i–k). However, we observed significant decreases in grain yield in *ppr035* when they were planted in Hainan (Supplementary Figure S16g). The observed yield penalty is mainly attributed to the decrease in fecundity (Supplementary Figure S16f), which is consistent with the detection of several known genes (e.g., *OsEMSA1*, *OsPRP1*) related to pollen development and fertility among DEGs (Supplementary Table S10). The reduction in fecundity in *ppr406* is not apparent, indicating that this should be attributed to RNA editing at *rps4*. In addition, as the field experiment was conducted in Hainan, where the rice plant may suffer a low temperature during winter. The reduced fecundity in *ppr035* may be also caused by the lower temperature once the abnormal RNA editing at SNV00404 (*rps4*) is associated with rice susceptibility to low temperature. However, this hypothesis is required further experimental validation.

### *PPR035* and *PPR406* Regulate Drought Tolerance Potentially Through RNA Editing at *orfX*

As *PPR035* and *PPR406* are both involved in RNA editing at *orfX*, we considered that they should regulate drought/salt tolerance through RNA editing at *orfX*. *orfX* was significantly upregulated in response to ABA, PEG-simulated osmotic stress, salinity, and oxidative stress (Supplementary Figure S17a). In contrast, *rps4* was mainly downregulated by high temperature, low temperature, ABA, osmotic stress, and oxidative stress (Supplementary Figure S17b). We detected many core WT-mutant DEGs, including *OCPI2*, which were also differentially expressed (Supplementary Table S11) between high and low expression genotypes (HEG and LEG) of *orfX*. Meanwhile, *orfX* had many ecotype-differentiated sites (Figure 1A). In addition, the expression of *orfX* suppressed the growth of *E. coli* under the salinity stress (Supplementary Figures S18a–d), indicating the negative roles of orfX in the salinity tolerance. All these results suggested that RNA editing at *orfX* is potentially associated with rice drought and salinity tolerance.

orfX contains several trans-membrane helixes that are important to its biological function. Based on the prediction, the matured (fully edited) and the intact (non-edited) orfX had many differences in trans-membrane helixes (Supplementary Figure S4). The non-synonymous sites edited by *PPR035* (SNV001178) and *PPR406* (SNV001187) can alter the amino acid which are located at the third trans-membrane helix (Supplementary Figure S4). It may have some influences on its normal function.

### Evolution of *PPR035* and *PPR406* in Wild Rice, Landraces, and Modern Cultivars

**Figure 5:**
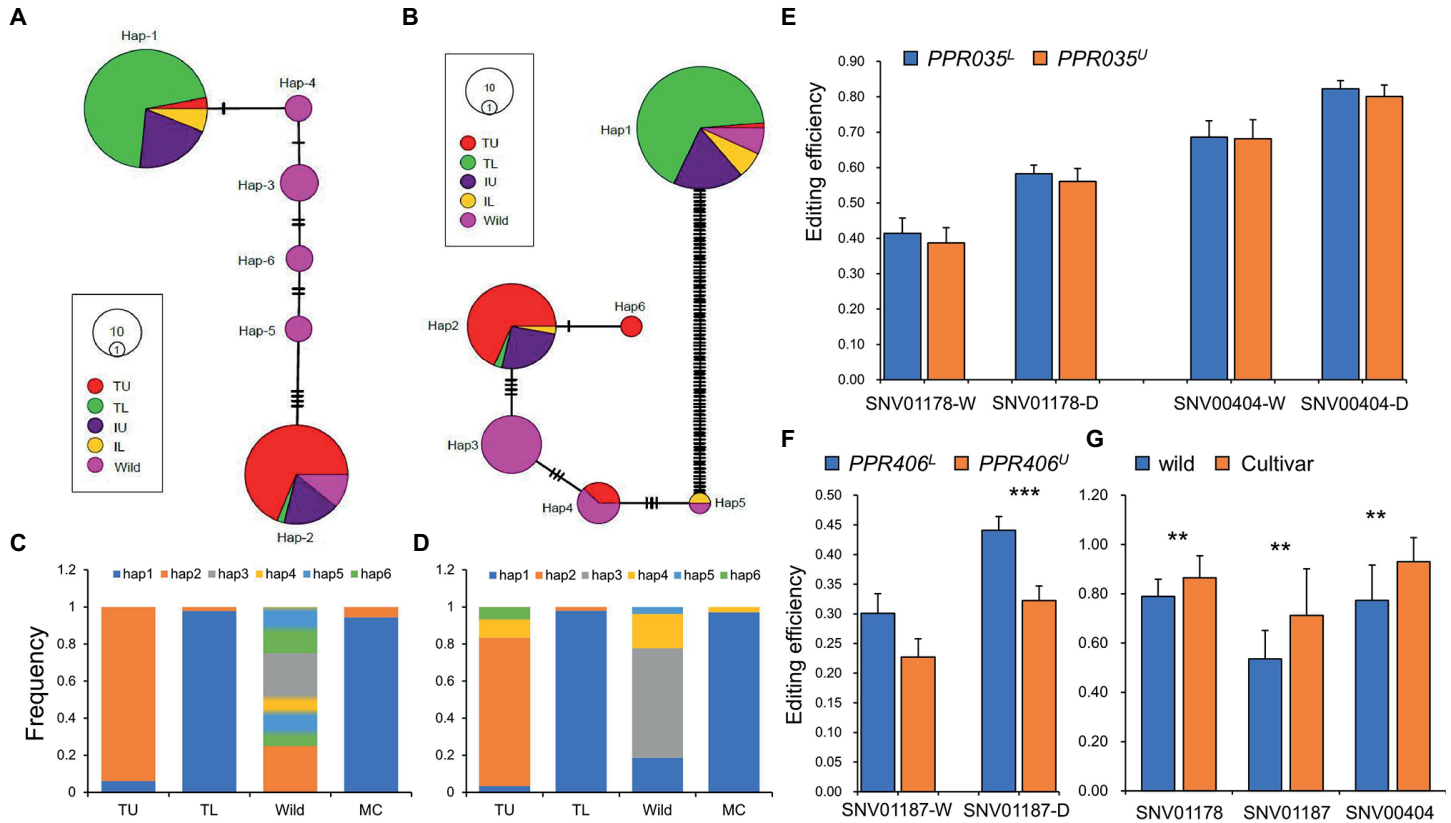
Haplotypes of PPR035 and PPR406 in wild, landraces, and modern cultivars. **(A)** Haplotype network of *PPR035* in wild rice, upland rice, and lowland rice. **(B)** Haplotype network of *PPR406* in wild rice, upland rice, and lowland rice. **(C)** The frequency of six major haplotypes of PPR035 in wild rice, landraces, and modern cultivars (MC). **(D)** The frequency of six major haplotypes of PPR406 in wild rice, landraces, and modern cultivars. **(E)** Editing efficiencies at the PPR035 regulating sites in upland- and lowland-dominant haplotypes (*PPR035^U^* and *PPR035^L^*). **(F)** Editing efficiencies at the PPR406 regulating site in upland- and lowland-dominant haplotypes (*PPR406^U^* and *PPR406^L^*). **(G)** Editing efficiencies at related sites in modern cultivar and wild rice. ^**^ and ^***^ indicate significance at *p* < 0.01 and *p* < 0.001 by independent *t*-test. IL, Intermediate lowland rice; TL, Typical lowland rice; IU, Intermediate upland rice; and TU, typical upland rice.

Upland rice experienced bi-directional selection during its domestication in upland agroecosystems (Xia et al., 2019). It is an ideal germplasm to identify beneficial alleles of a drought-resistant gene. *PPR035* and *PPR046* are located in the genomic region under balancing selection in upland rice (Supplementary Table S12), indicating their potential roles in drought adaptation. *PPR035*, which has ten SNPs with one missense SNP called from resequencing (Supplementary Figure S2a, Supplementary Table S13), forms six major haplotypes (Figure 5A). *PPR406* had 106 SNPs with 24 missense SNPs (Supplementary Figure S2b, Supplementary Table S13) and formed six major haplotypes (Figure 5B). Based on their frequencies in typical upland and lowland rice (Figures 5C,D), Hap-1 of *PPR035* and *PPR406* could be determined as the lowland-dominant allele (coded as *PPR035^L^* and *PPR406^L^*), whereas Hap-2 of *PPR035* and *PPR406* were upland-dominant (coded as *PPR035^U^* and *PPR406^U^*). *PPR035^U^* is a wild rice-derived allele that can be detected in wild rice, whereas *PPR035^L^* is possibly a novel mutation obtained during lowland rice domestication as it is absent in wild rice. In contrast, *PPR406^L^* should be derived from its wild ancestor, whereas *PPR406^U^* could be a novel mutation during upland rice domestication. It is noteworthy that RNA editing efficiencies at the three sites regulated by *PPR035* and *PPR406* were significantly lower in common wild rice than those in modern cultivars (Figure 5G).

Both *PPR035^U^* and *PPR406^U^* confer missense SNPs in their functional motifs (Supplementary Figures S2a,b; Supplementary Table S13), which may affect their protein structures (Supplementary Figures S2c–d). However, we could only detect significant differences in RNA editing efficiency at SNV001187 between genotypes of *PPR406^U^* and *PPR406^L^* under drought conditions (Figures 5E,F). We believe that the significantly higher expression of *PPR035^U^* (Supplementary Table S14) in the typical upland rice may compensate for alterations in the amino sequences. In addition, many of the core DEGs showed great expression divergence between genotypes conferring *PPR035^U^*/*PPR406^U^* and *PPR035^L^*/*PPR406^L^* (Supplementary Table S14). Given the dominance of *PPR035^U^* and *PPR406^U^* in the typical upland rice which performed better in the drought-treated field (Supplementary Table S15), we considered them beneficial alleles of drought tolerance and may have minor penalties in rice productivity. However, the frequencies of *PPR035^U^* and *PPR406^U^* in modern cultivars were very low (Figures 5C,D), indicating their great potential for utilization in rice breeding.

## Discussion

### PPR Genes Are in Response to Several Abiotic Stresses and Phytohormones

PPR proteins in plants are a large family for organelle RNA editing (Lurin et al., 2004; Fujii and Small, 2011; Hammani and Giegé, 2014; Yan et al., 2018). Based on the transcriptomic investigation, we found that most rice PPR genes responded to abiotic stresses and/or phytohormones, particularly for the mitochondrion-located DYW PPR genes. Meanwhile, many PPR genes, including *PPR035* and *PR406*, are downregulated under treatments of osmotic, salinity, and oxidative stresses, indicating that they may potentially act as negative regulators for rice adaptation to adverse environments.

### Mitochondrial RNA Editing Impacts on Rice Drought Tolerance and Productivity

Mitochondrial RNA editing is required to develop mitochondria, which play a crucial role in normal plant life activities. Defects in PPR gene-mediated mitochondrial RNA editing are always associated with the dysfunction of mETC (Sosso et al., 2012; Zhu et al., 2012, 2014), growth retardation (Yuan and Liu, 2012; Liu et al., 2013), male sterility (Xiao et al., 2018; Zhang et al., 2020), and environmental adaptation (Plessis et al., 2011; Murayama et al., 2012; Sechet et al., 2015; Emami and Kempkenm, 2019). In this study, we investigated rice genome-wide mitochondrial RNA editing under the well-watered and drought-treated conditions. We observed the genotype-specific and site-specific manners of RNA editing in response to drought, which means that some genotypes/sites upregulate while other genotypes/sites downregulate their editing efficiencies when they encounter drought. Based on the results of the correlation analysis, we have built potential associations of mitochondrial RNA editing with drought tolerance and/or agronomically important traits. A tradeoff exists in PPR gene-mediated mitochondrial RNA editing, particularly editing genes encoding proteins of the mETC complex, between drought tolerance and productivity. This observation is consistent with many previous reports in which the knockout of a PPR gene related to mETC could cause pleiotropic phenotypes in growth and tolerance to abiotic stresses (Yuan and Liu, 2012; Zhu et al., 2012, 2014). We also identified many sites whose editing efficiencies differed between upland and lowland rice. Given the two rice ecotypes domesticated in agroecosystems under contrasting soil-water conditions (International Rice Research Institute (IRRI), 1975; Xia et al., 2019), these ecotype-differentiated sites and their regulators may contribute to rice adaptation to drought stress.

### *PPR035* and *PPR406* Negatively Regulate Rice Drought Tolerance

*PPR035* and *PPR406* are the highly differentiated genes between the upland and lowland rice ecotypes. They are involved in RNA editing at *orfX* and/or *rps4*, which contain several ecotype-differentiated editing sites. In previous reports, the RNA editing defects at *rps4* and *orfX* resulted in diverse plant phenotypes, including embryonic lethality, early flowering, growth retardation, and male sterility (Supplementary Table S16). We found that RNA editing regulated by *PPR035* and *PPR406* at *rps4* and *orfX* was potentially associated with drought tolerance. The upregulation of several drought-tolerant DEGs (e.g., *OCPI1*, *OsLEA3*) in *ppr035* and *ppr406* can explain its improved drought/salt tolerance partially by osmotic adjustment.

The plant phenotype induced by mitochondria relies on the retrograde signal, which forms in mitochondria, passes *via* the endoplasmic reticulum (ER), and finally regulates gene expression in the nucleus (Rhoads and Subbaiah, 2007; Wang et al., 2020). *PPR035 and PPR406* is likely to followed this retrograde signal mode (mitochondrion-ER-nucleus) based on the core DEGs detected in *ppr035* and *ppr406*. The widely reported retrograde signals induced by mitochondrial dysfunction are the upregulation of *AOXs* and/or ROS burst (Rhoads and Subbaiah, 2007; Huang et al., 2016). However, we did not detect the two common signals in *ppr035* and *ppr406*. We hypothesis that RNA editing at *orfX* is associated with drought tolerance in rice. First, *orfX* expression was induced by drought and salt (Supplementary Figure S17a). Second, many core DEGs showed significant differences between the HEGs and LEGs of *orfX* (Supplementary Table S11). Third, the overexpression of *orfX* in *E. coli* suppressed its growth in salinity stress. The non-synonymous editing sites regulated by PPR035 and PPR406 locate both at the third trans-membrane helix, indicating this structure is very important to orfX. However, the role played by orfX in rice drought tolerance and its molecular mechanism require further investigation.

### Natural Variants of *PPR035* and *PPR406* in Upland Rice Have Potentials to Improve Drought Tolerance in Rice

In the rainfed drought-prone upland agroecosystem, upland rice was subjected to bi-directional selection between drought tolerance and productivity, resulting in balanced polymorphism in drought tolerance and productivity related genomic region (Xia et al., 2019). The genomic regions of *PPR035* and *PPR406* are only under balancing selection in upland rice. We further identified natural variants of the two genes in upland rice, which were highly differentiated from their dominant haplotypes in lowland rice. Many core DEGs, which are potentially associated with drought tolerance, possess expression divergence between upland and lowland rice, indicating that *PPR035* and *PPR406* contribute to the adaptation of upland rice to the drought-prone upland agroecosystem.

The most limitation to utilize PPR genes in breeding is the unwanted pleiotropic effects, such as growth retardation and reproductive failure, by PPR-mediated mitochondrial RNA editing at the mETC (Toda et al., 2012; Liu et al., 2013; Xiao et al., 2018; Li et al., 2019; Zhang et al., 2020). In this study, we observed improved tolerance to drought and salinity in *ppr406* without growth retardation and yield penalty. Given *PPR406* regulates RNA editing at *orfX*, it is possible for us to utilize PPR genes that are not related to RNA editing at mETC. Meanwhile, performances of landraces conferring *PPR035*^U^ and *PPR406*^U^ also provide positive indications, as they represent improved drought tolerance without any significant decreases in fertility. This result suggests that *PPR035*^U^ and *PPR406*^U^ are beneficial alleles during upland rice domestication and have great potential for rice improvement of drought tolerance.

## Data Availability Statement

The datasets presented in this study can be found in online repositories. The names of the repository/repositories and accession number(s) can be found at: https://www.ncbi.nlm.nih.gov/, PRJNA306542; https://www.ncbi.nlm.nih.gov/, PRJNA609211; https://www.ncbi.nlm.nih.gov/, PRJNA260762; and https://www.ncbi.nlm.nih.gov/, PRJNA825139.

## Author Contributions

HX and LL designed the experiments. JX, HX, ZL (Zhi Luo), GH, HZ, JL, and ZL (Zhaoyang Li) conducted the field and molecular experiments. HX, JX, and LW analyzed the data. ZL (Zhi Luo), JX, and TL prepared the plant materials used in this study. HX, JX, ZL (Zhi Luo), and LL wrote the manuscript. All authors contributed to the article and approved the submitted version.

## Funding

This work is supported by Shanghai Natural Science Foundation (20ZR1449300), Shanghai Agriculture Applied Technology Development Program (G2016060107), National Key Research and Development Program of China (2018YFE0106200), Shared Platform of Crop Germplasm Resources in Shanghai (18DZ2293700), and Platform for National Crop Germplasm Resources (Shanghai; NICGR2021-2).

## Conflict of Interest

The authors declare that the research was conducted in the absence of any commercial or financial relationships that could be construed as a potential conflict of interest.

## Publisher’s Note

All claims expressed in this article are solely those of the authors and do not necessarily represent those of their affiliated organizations, or those of the publisher, the editors and the reviewers. Any product that may be evaluated in this article, or claim that may be made by its manufacturer, is not guaranteed or endorsed by the publisher.

## Footnotes

^1^ http://www.usadellab.org/cms/uploads/supplementary/Trimmomatic

^2^ https://ccb.jhu.edu/software/hisat2/index.shtml

^3^ https://htseq.readthedocs.io/en/release_0.11.1/

^4^ http://www.bioconductor.org/packages/release/bioc/html/edgeR.html/

^5^ http://elder.njjcbio.com/html_en/search.php

^6^ www.cbs.dtu.dk/services/TargetP/

^7^ https://www.ncbi.nlm.nih.gov/Structure/cdd/wrpsb.cgi?seqinput=YP_002000561.1

^8^ http://bioinf.cs.ucl.ac.uk/psipred/
