# Supplementary material for: Pentatricopeptide Repeat Gene-Mediated Mitochondrial RNA Editing Impacts on Rice Drought Tolerance
Source: Front Plant Sci. 2022 Jul 19;13:926285. doi: 10.3389/fpls.2022.926285 (PMC9343880; doi:10.3389/fpls.2022.926285)
Supplement: Supplementary file 4 [file Data_Sheet_4.pdf]

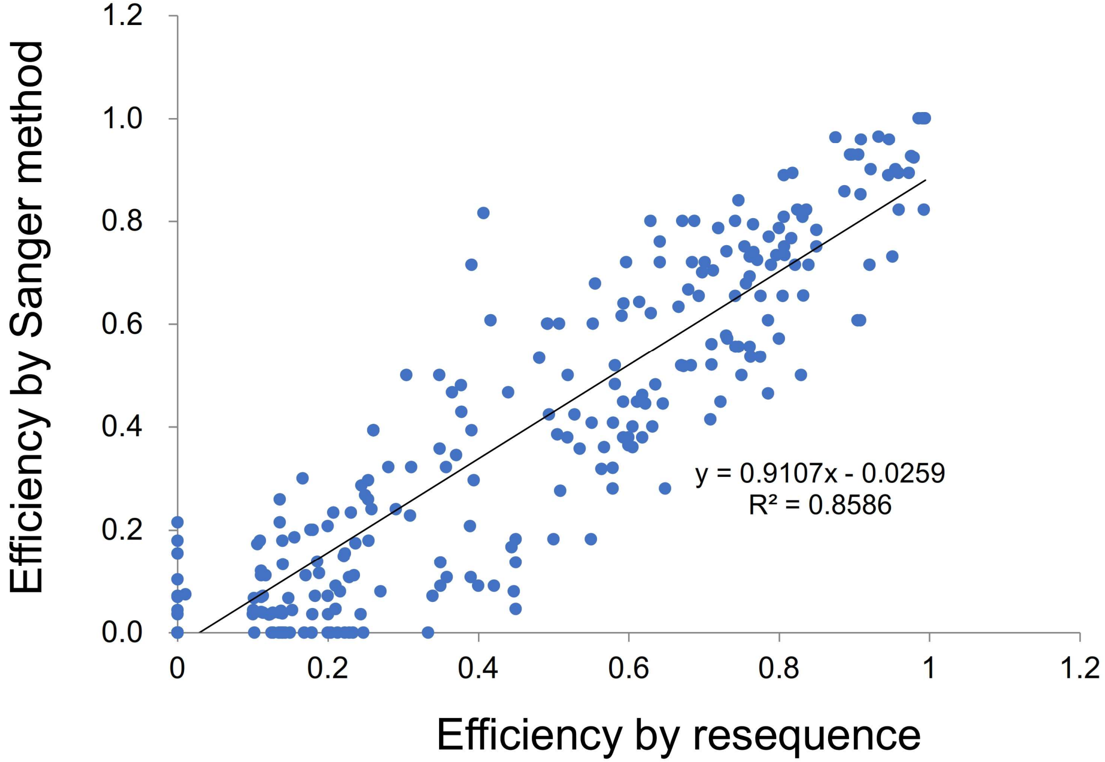

Figure S1. Correlation of RNA editing efficiencies quantified by high-throughput sequencing and the Sanger method.

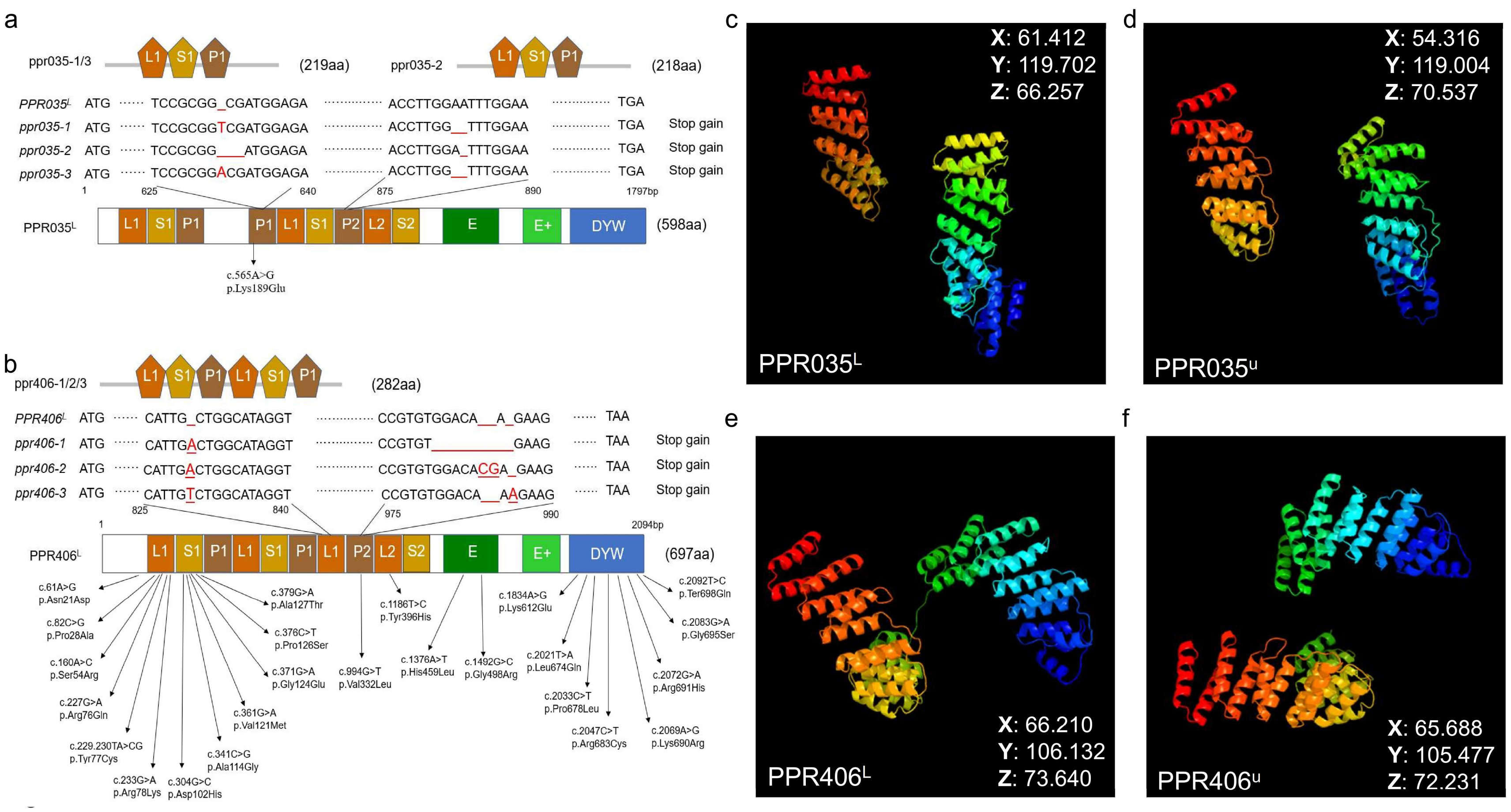

Figure S2. Variants in DNA sequences and protein structures of *PPR035* (**a**) *and PPR406* (**b**) in knockout mutants, typical lowland rice, and typical upland rice. **c.** The protein structure of PPR035^{L} and PPR035^{U} predicted by Phyre2. **d.** The protein structure of PPR406^{L} and PPR406^{U} predicted by Phyre2.

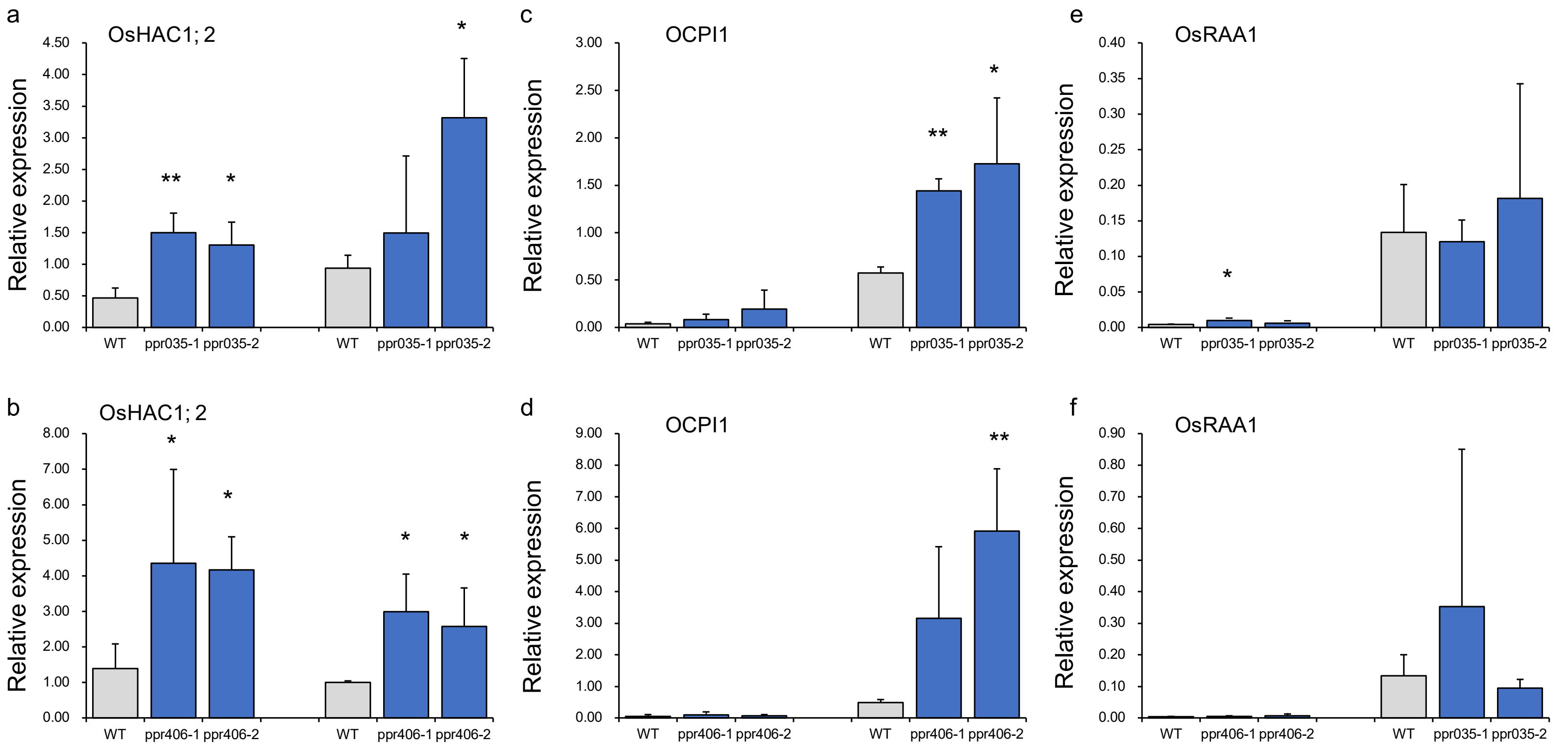

Figure S3. Validation of three differentially expressed genes identified by qPCR. Three biological replicates are used in qPCR. *ACTIN1* is used as the reference.

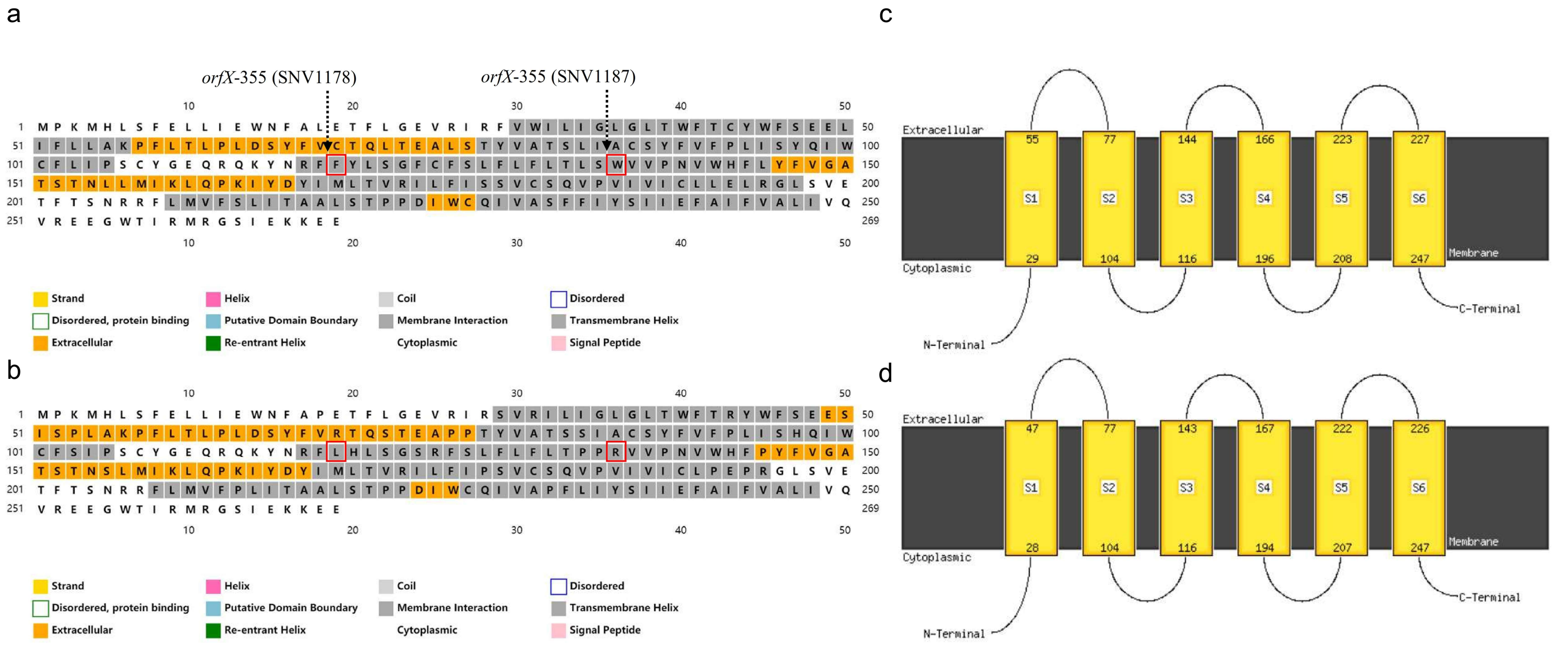

Figure S4. The amino sequence and structure of orfX. **a.** The amino sequence of matured (fully-edited) orfX. **b.** The amino sequence of intact (non-edited) orfX. **c.** Transmembrane helices predicted in matured orfX. **d.** Transmembrane helices predicted in intact orfX. The amino acid in red box is modified by PPR035 or PPR406.

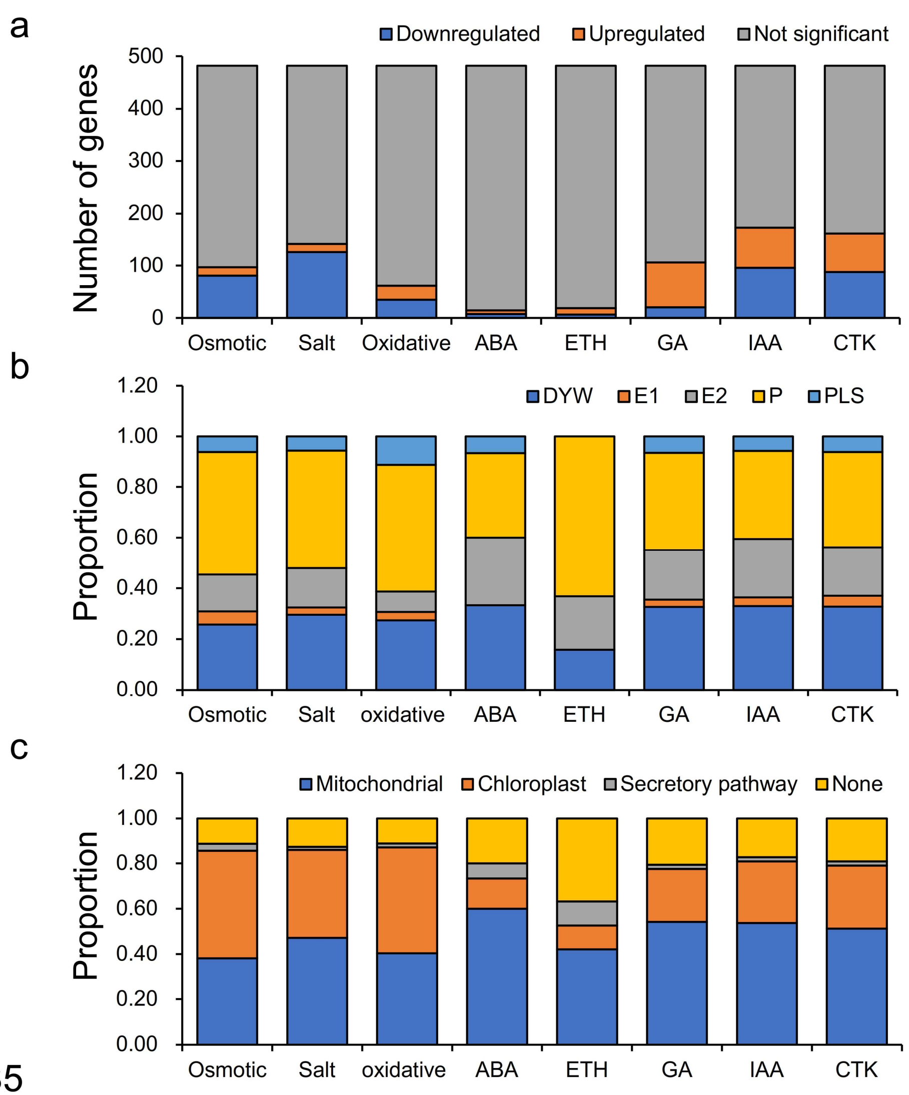

Fig. S5

Figure S5. PPR genes in responses to various abiotic stresses and phytohormones. **a.** Number of downregulated and upregulated PPR genes in responses to stresses and phytohormones. **b.** Proportions of PPR genes in different types in responses to stresses and phytohormones. **c.** Proportions of PPR genes with different subcellular localization in responses to stresses and phytohormones. ABA: abscisic acid; ETH: ethylene; GA: gibberellin; IAA: auxin; CTK: cytokinin.

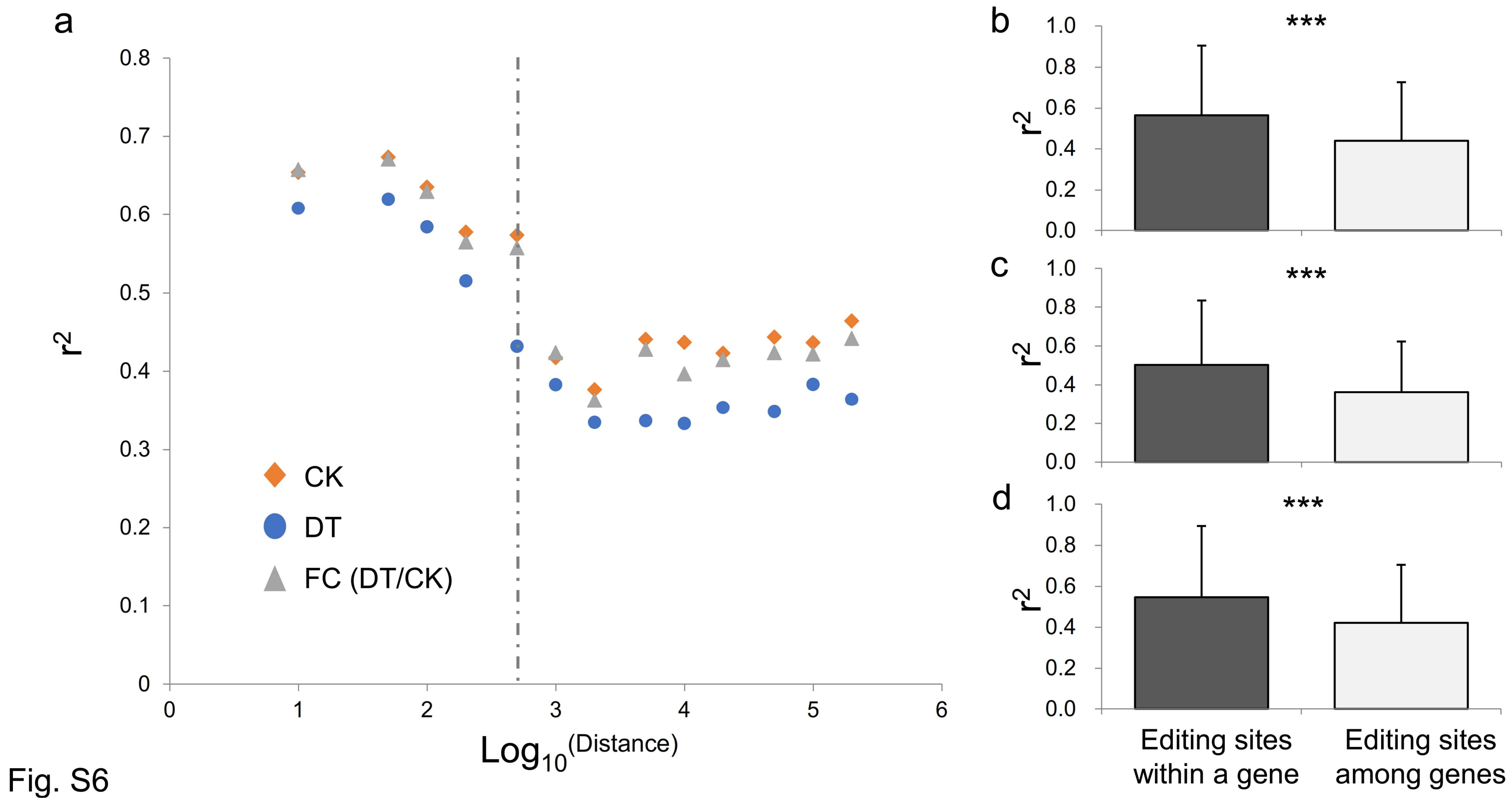

Figure S6. Correlations (Pearson's correlation coefficients, r^2) of RNA editing efficiencies among sites. **a.** Averaged r^2 of RNA editing efficiencies among sites with different distances. **b.** Averaged r^2 of RNA editing efficiencies among sites within or not within a same mitochondrial gene in the well-watered (CK) field. **c.** Averaged r^2 of RNA editing efficiencies among sites within or not within a same mitochondrial gene in the drought-treated (DT) field. **d.** Averaged r^2 of fold change from CK to DT of editing efficiencies among sites within or not within a same mitochondrial gene.

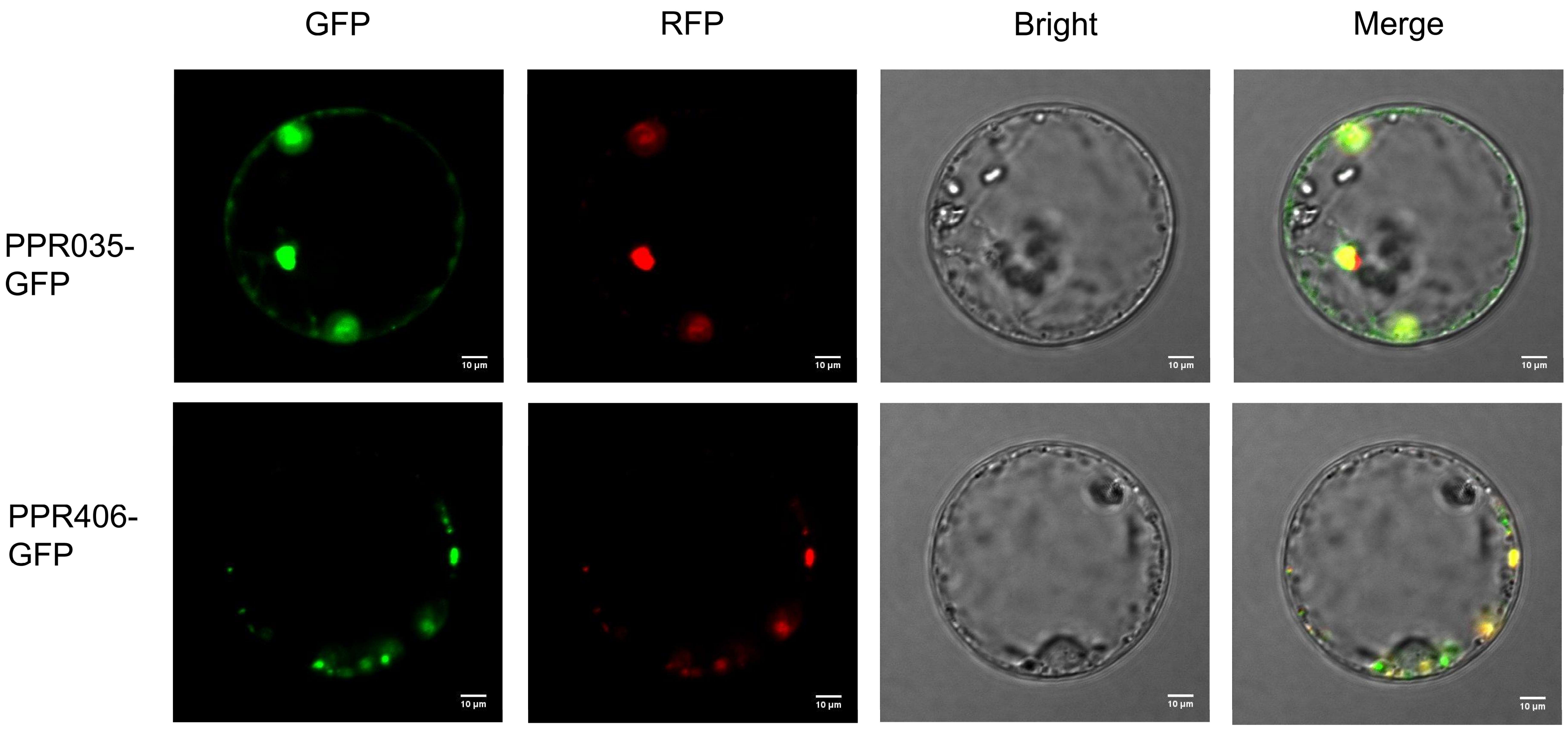

Figure S7. Subcellular localization of two PPR proteins in rice protoplast. mstp-RFP was used as the mitochondrion indicator.

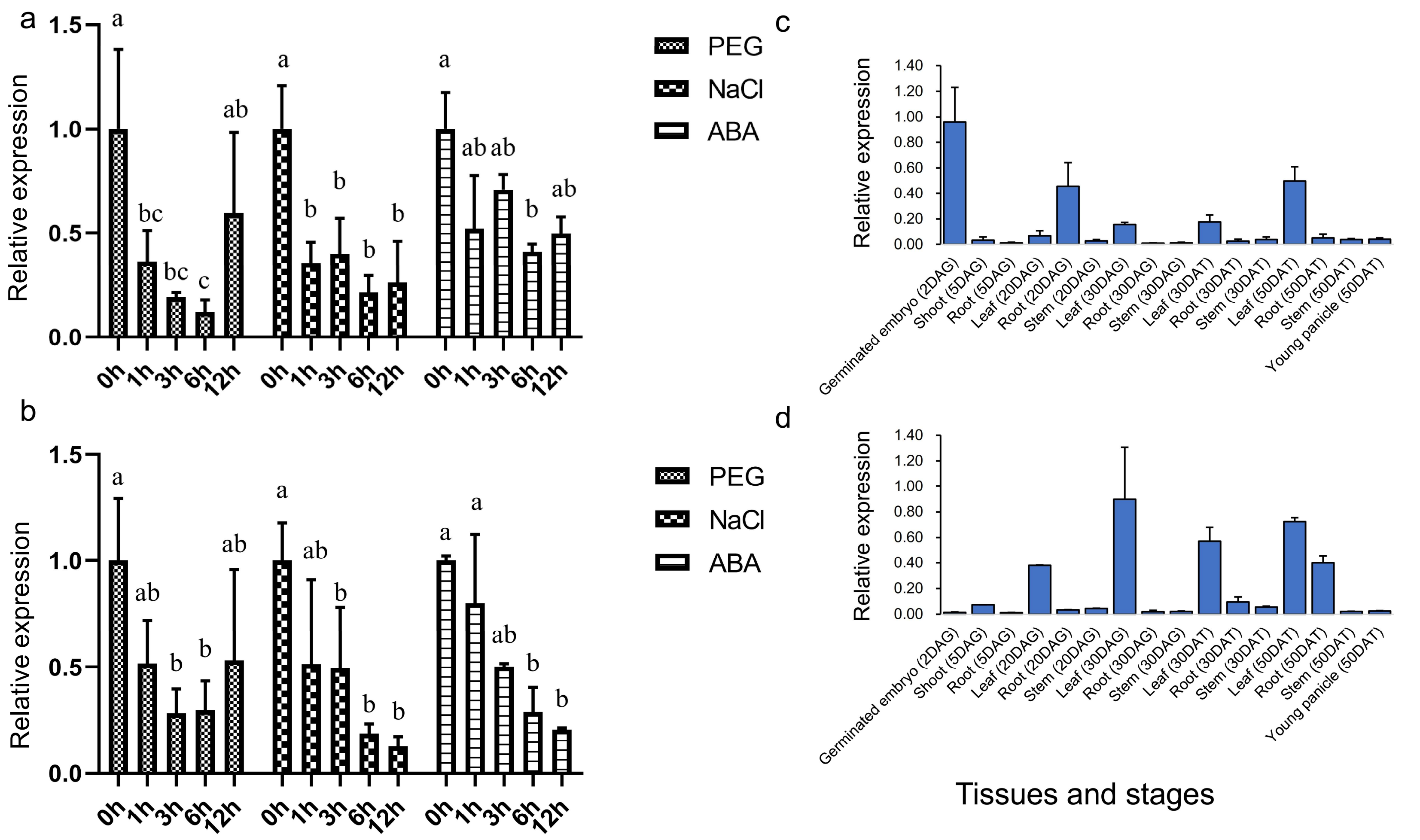

Figure S8. Expressions of *PPR035 and PPR406* in wild type (WT, Nipponbare) quantified by qPCR. **a, b.** Expressions of *PPR035* (a) *and PPR406* (b) in treatments of ABA and various stress. **c, d.** Expressions of *PPR035* (c) *and PPR406* (d) in different tissues at different growth stages. *ACTIN1* is used as the reference.

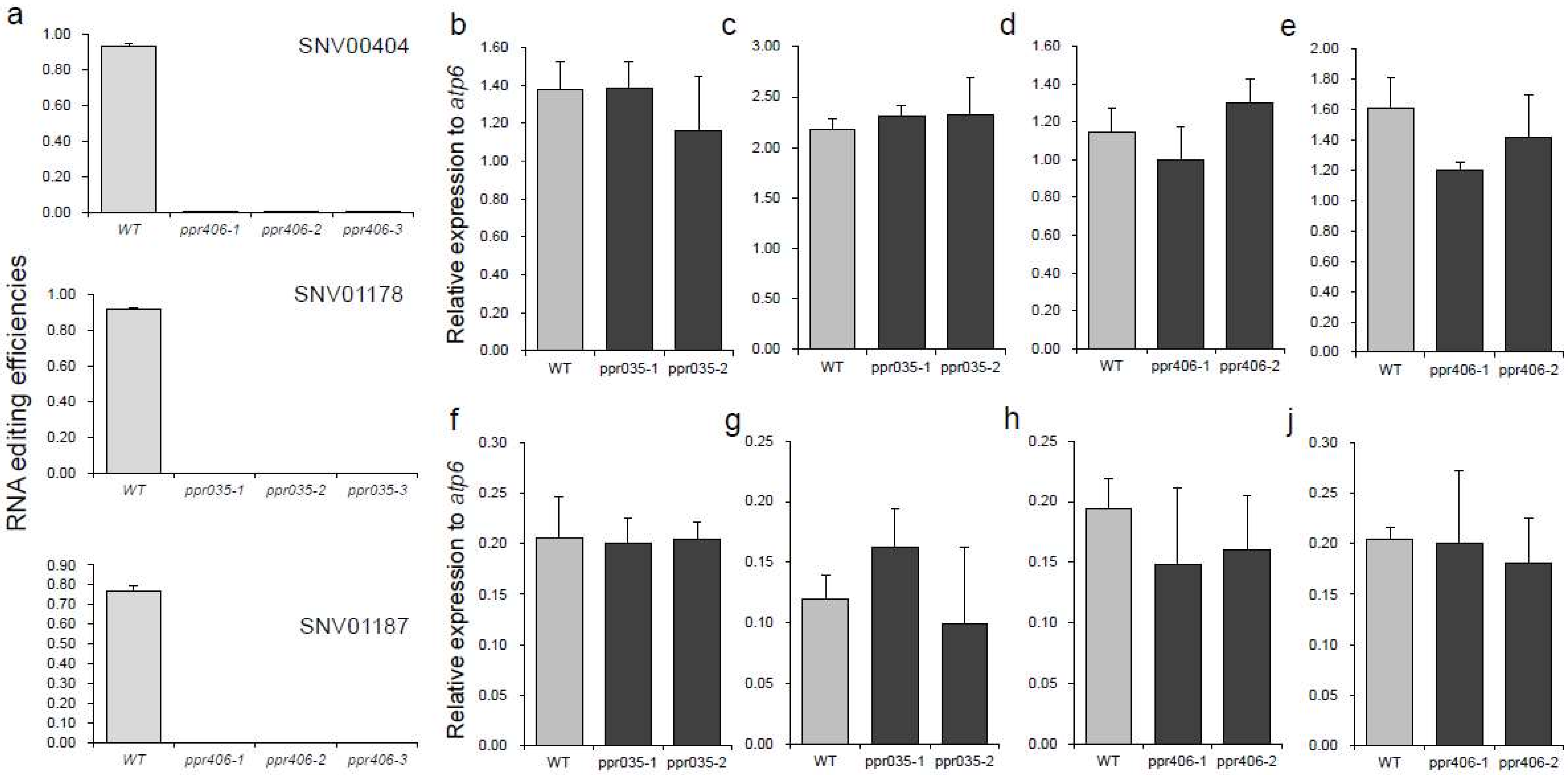

Figure S9. RNA editing efficiencies at two sites (**a**) and expressions of *orfX* (**b-e**) and *rps4* (**f-j**) in wild type (WT, Nipponbare) and knockout mutants (*ppr035* and *ppr406*) under normal (**b, d, f, h**) and PEG-simulated osmotic conditions (**c, e, g, i**). *ATP6* is used as the reference.

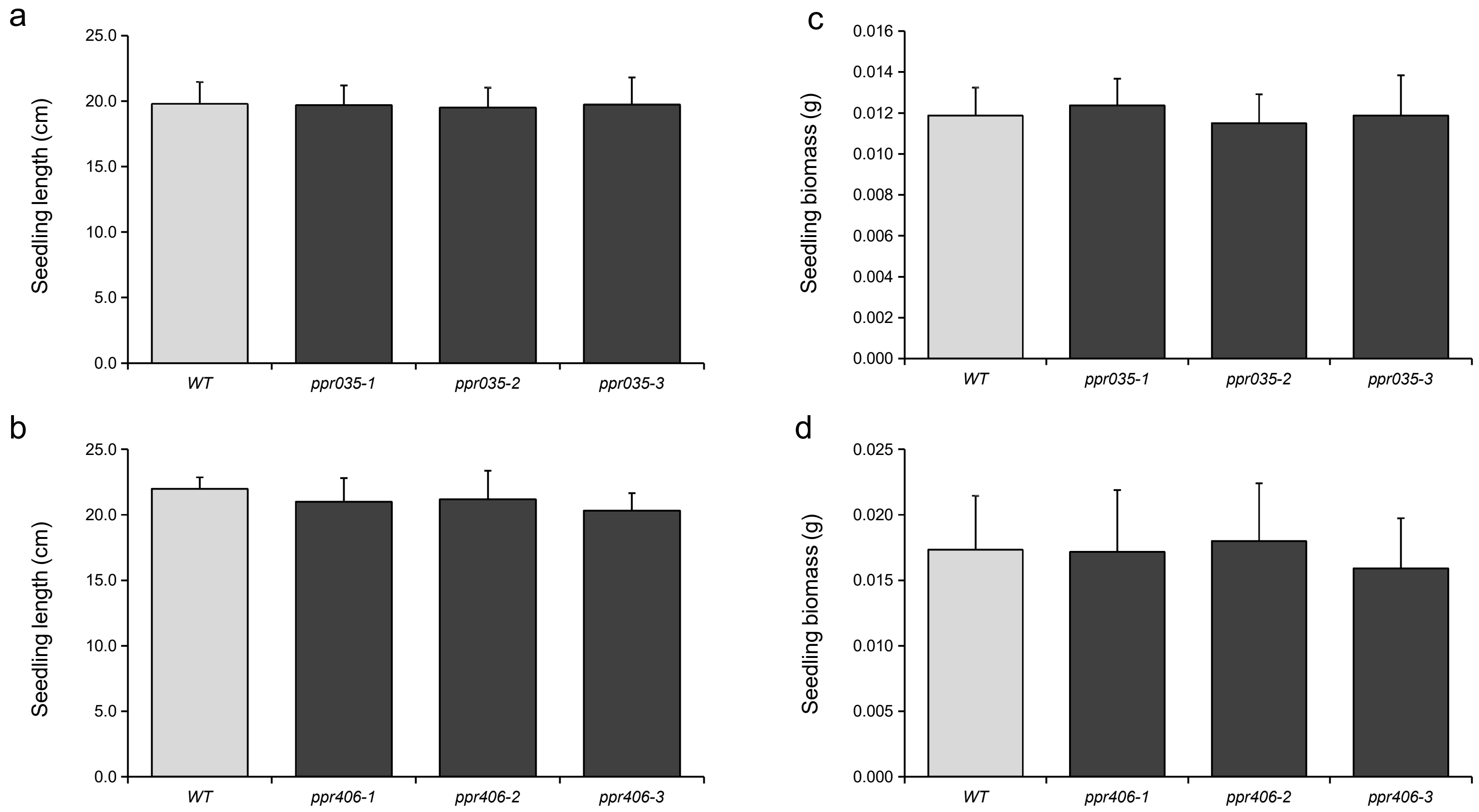

Figure S10. The seedling length and biomass of the wild type (WT) and mutants (*ppr035*-1/2/3 and *ppr406*-1/2/3) before the treatment of 20% PEG6000 simulated osmotic stress. a. Seedling length measured from WT and *ppr035*-1/2/3. b. Seedling length measured from WT and *ppr406*-1/2/3. c. Seedling biomass measured from WT and *ppr035*-1/2/3. d. Seedling biomass measured from WT and *ppr406*-1/2/3. Seedling length and biomass were measured from eight individuals.

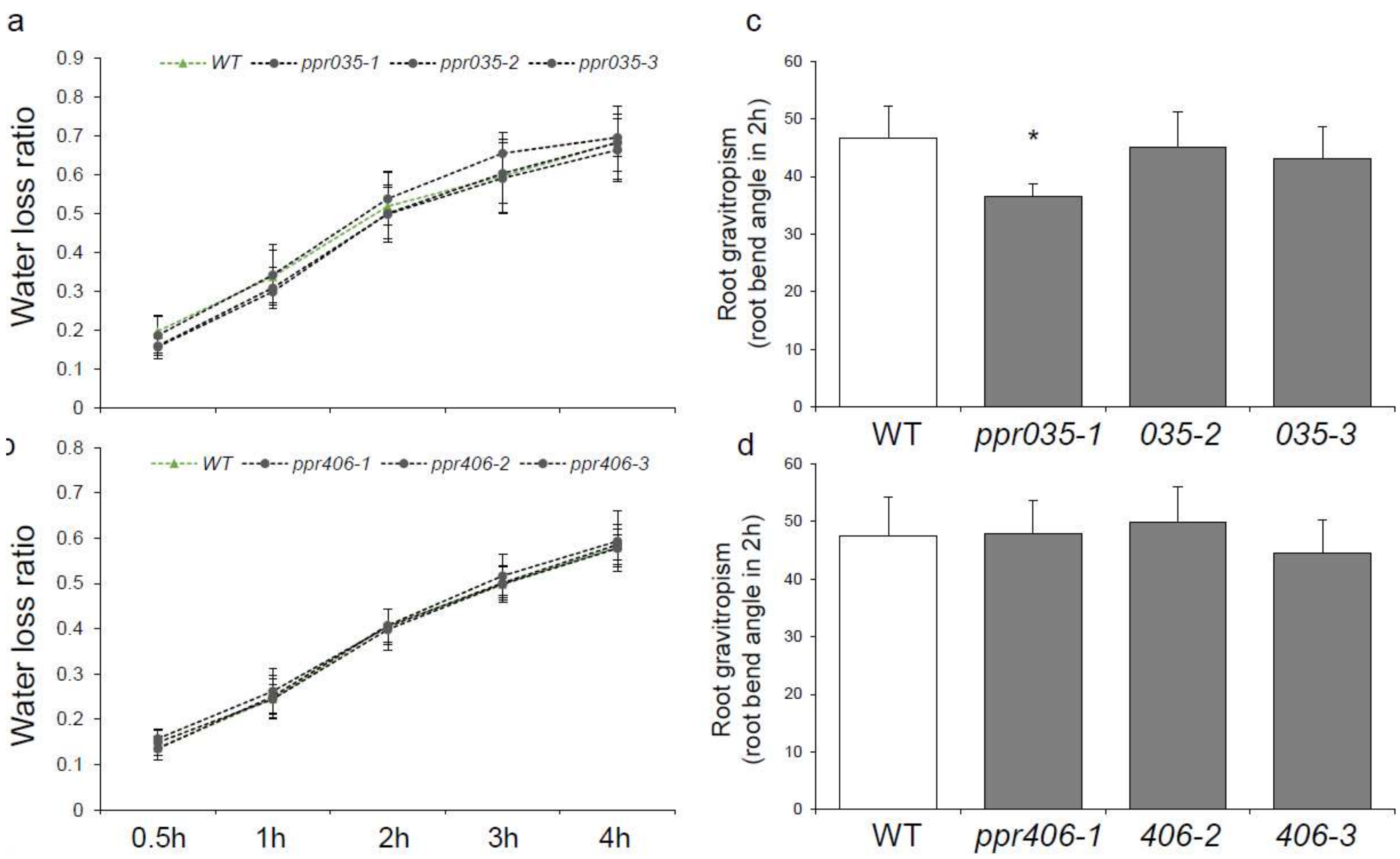

Figure S11. Traits of drought-avoidance measured in wild type (WT) and knockout mutants (*ppr035*-1/2/3 and *ppr406*-1/2/3). **a.** Rate of excised-leaf water loss (RWL) in WT and *ppr035*. **b.** RWL in WT and *ppr406*. **c**. Root gravitropism in in WT and *ppr035*. **d**. Root gravitropism in in WT and *ppr406*.

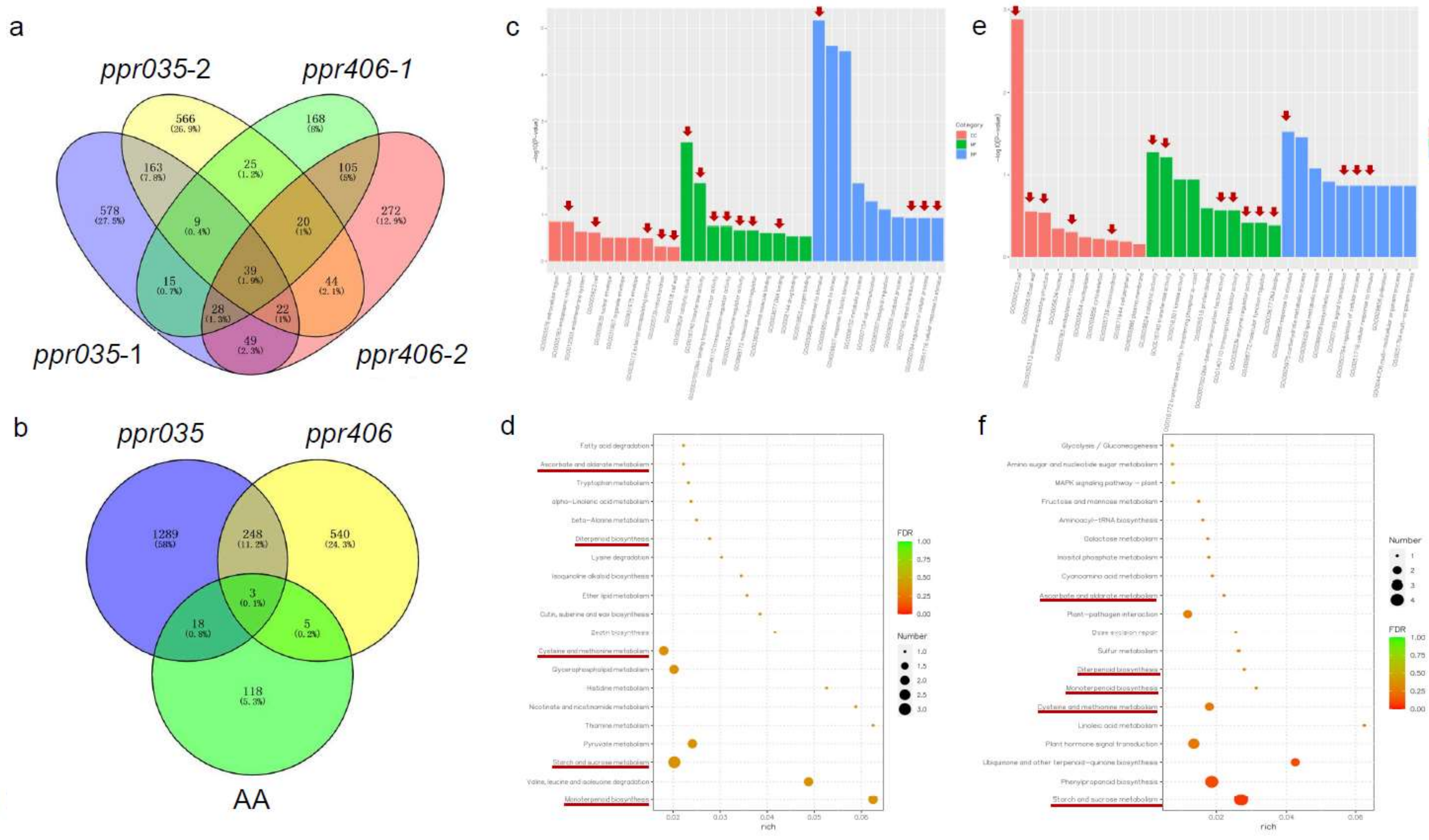

Figure S12. Transcriptomic impacts brought by the knockout of *PPR035 and PPR046*. **a.** The Venn diagram of differentially expressed genes (DEGs) detected between four knockout mutants (*ppr035*-1/2 and *ppr406*-1/2) and wild type. **b.** The Venn diagram of DEGs from *ppr035*, *ppr406*, and Antimycin A (AA) treatment (compared with normal growth condition). **c, e.** Top 10 GO terms in cell component (pink column), molecular function (green column), and biological process (blue column) enriched by DEGs of *ppr035* (**c**) and *ppr406* (**e**). Common GO terms are indicated by red arrows. **d, f.** Top 20 KEGG pathways enriched by DEGs of *ppr035* (**d**) and *ppr406* (**g**). Common pathways are red underlined.

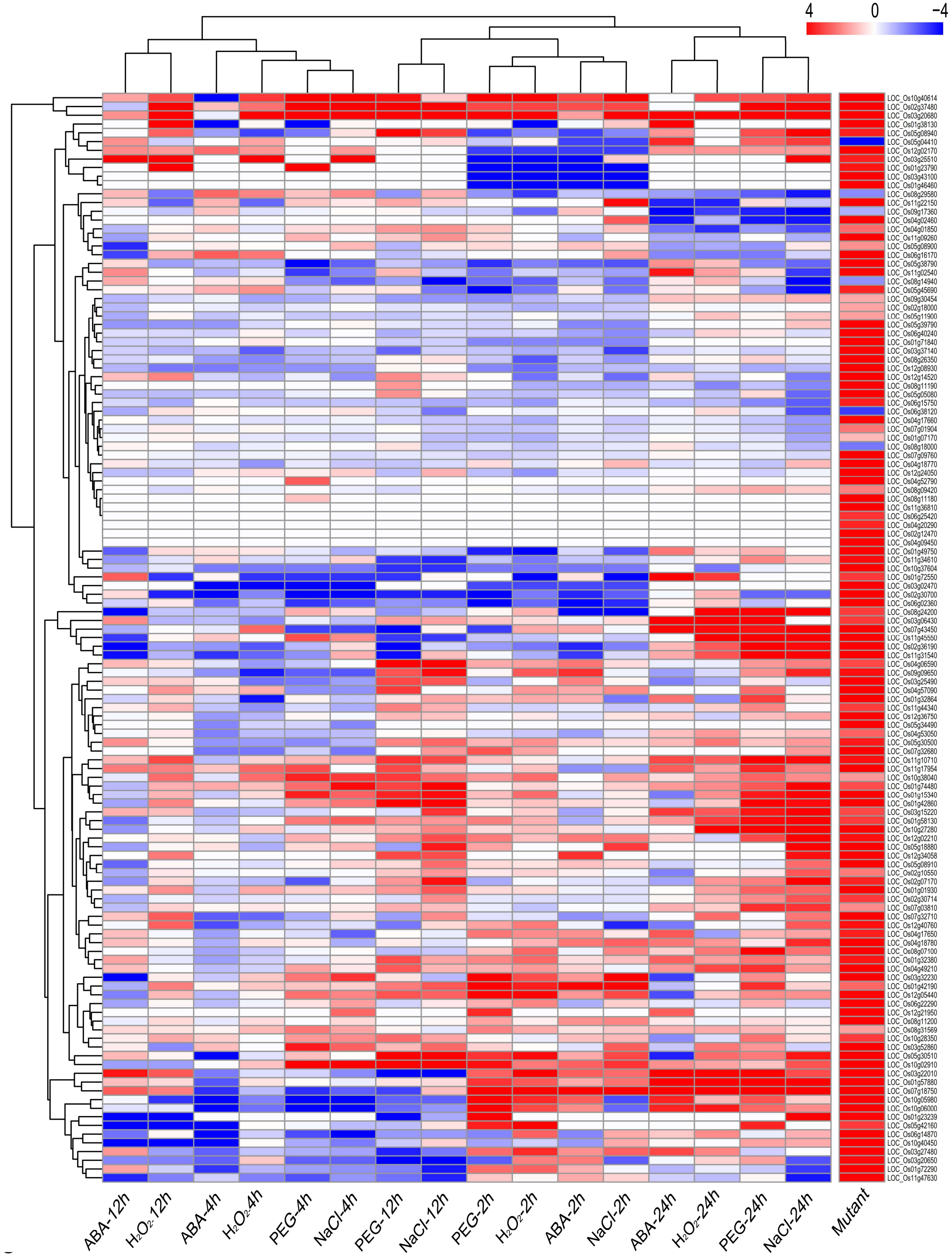

Figure S13. Expressions of core differentially expressed genes between mutants and the wild type in responses to ABA, salinity stress, PEG-simulated osmotic stress, and oxidative stress.

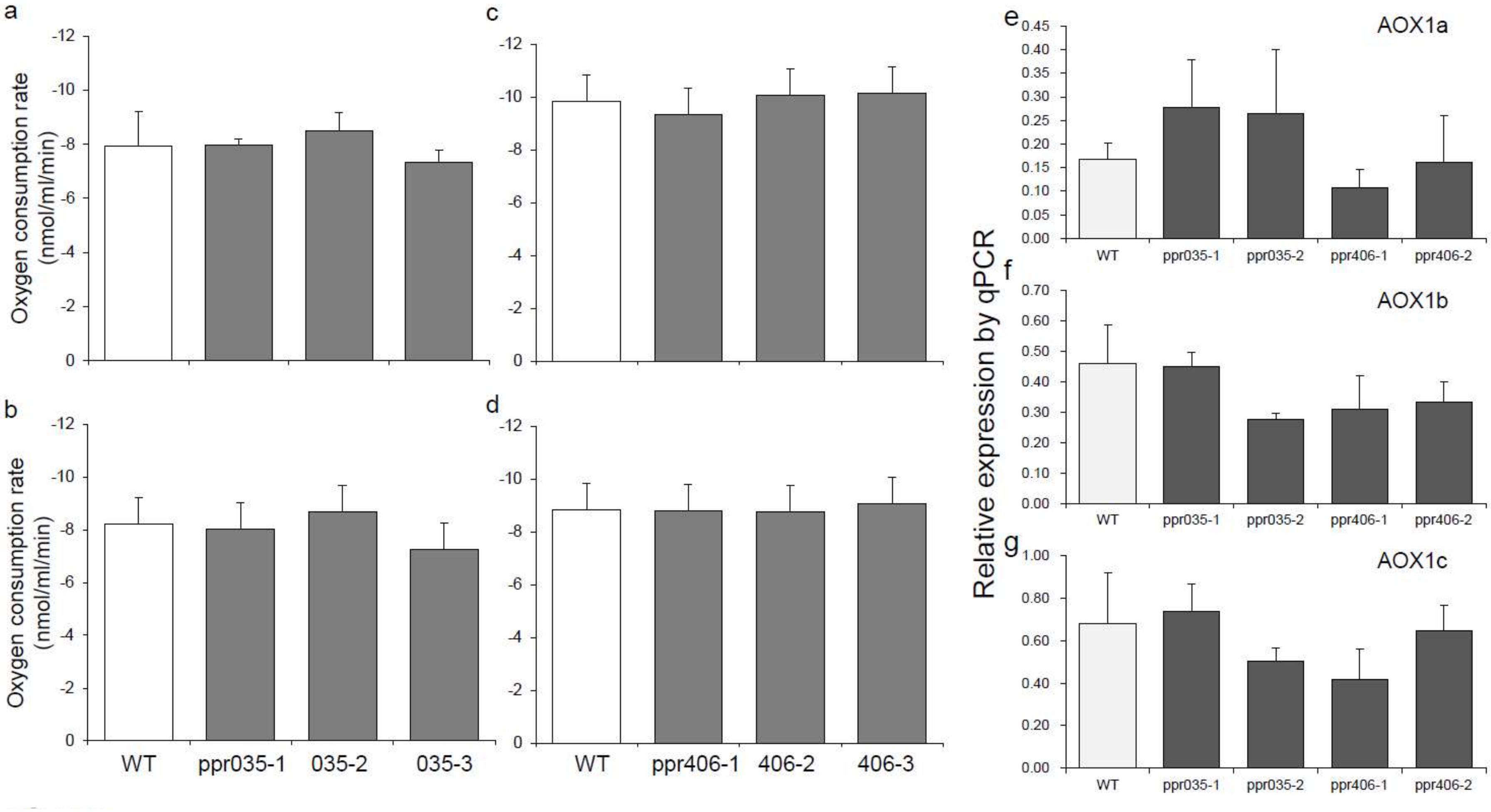

Figure S14. Impact of *PPR035 and PPR046* on respiration. **a-d**. Oxygen consumption rate measured in leaf samples of the wild type (WT) and knockout mutants (*ppr035*-1/2/3 and *ppr406*-1/2/3) under the normal condition (**a, c**) and PEG-simulated osmotic stress (**b, d**). **e-g.** Relative expression of *AOXs* in the wild type (WT) and knockout mutants (*ppr035*-1/2 and *ppr406*-1/2) under the normal condition. ACTIN1 is used as the reference.

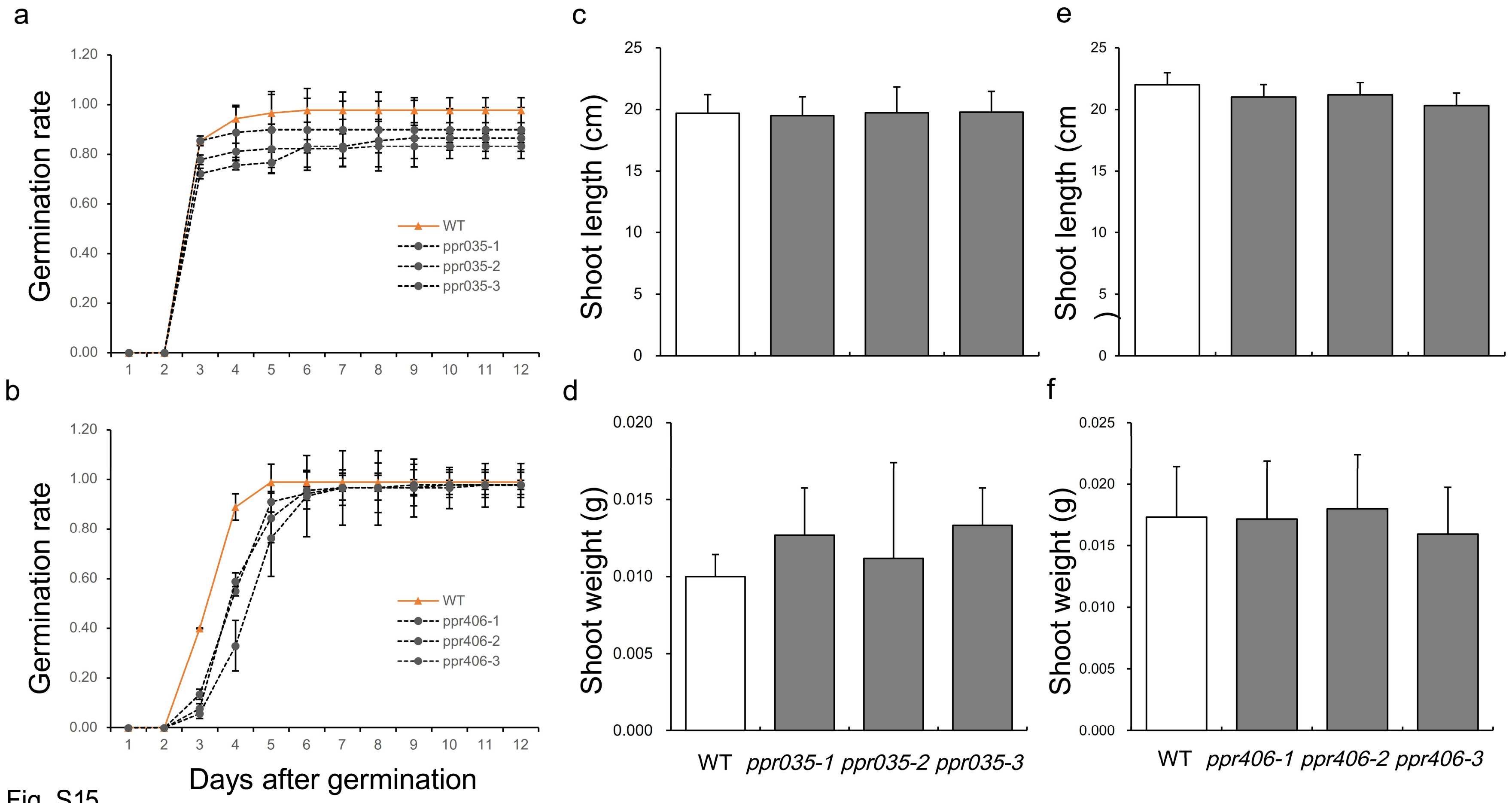

Fig. S15

Figure S15. Germination and early growth of the wild type (WT) and knockout mutants (*ppr035*-1/2/3 and *ppr406*-1/2/3). **a**. Germination of WA and *ppr035*-1/2/3. **b**. Germination of WT and *ppr406*-1/2/3. **c, d.** Shoot length and dry weight of two-week-old seedlings of WT and *ppr035*-1/2/3. **e, f.** Shoot length and dry weight of two-week-old seedlings of WT and *ppr406*-1/2/3.

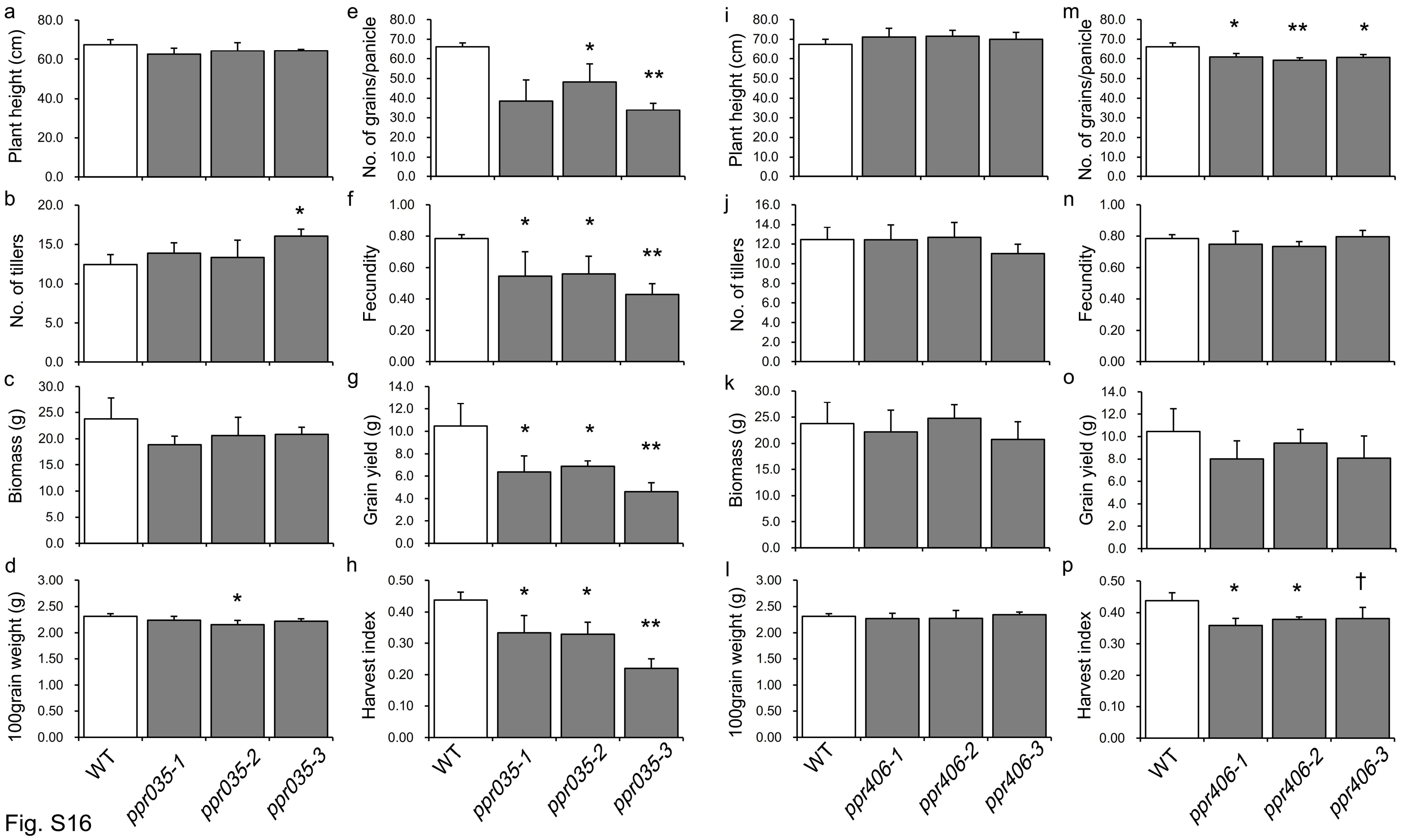

Figure S16. Field performance of wild type (WT, Nipponbare), *ppr035* (**a-h**) and *ppr406* (**i-p**). †, *, and ** indicate significant differences between WT and mutants at $p<0.1$, $p<0.05$, and $p<0.01$ by independent *t* test.

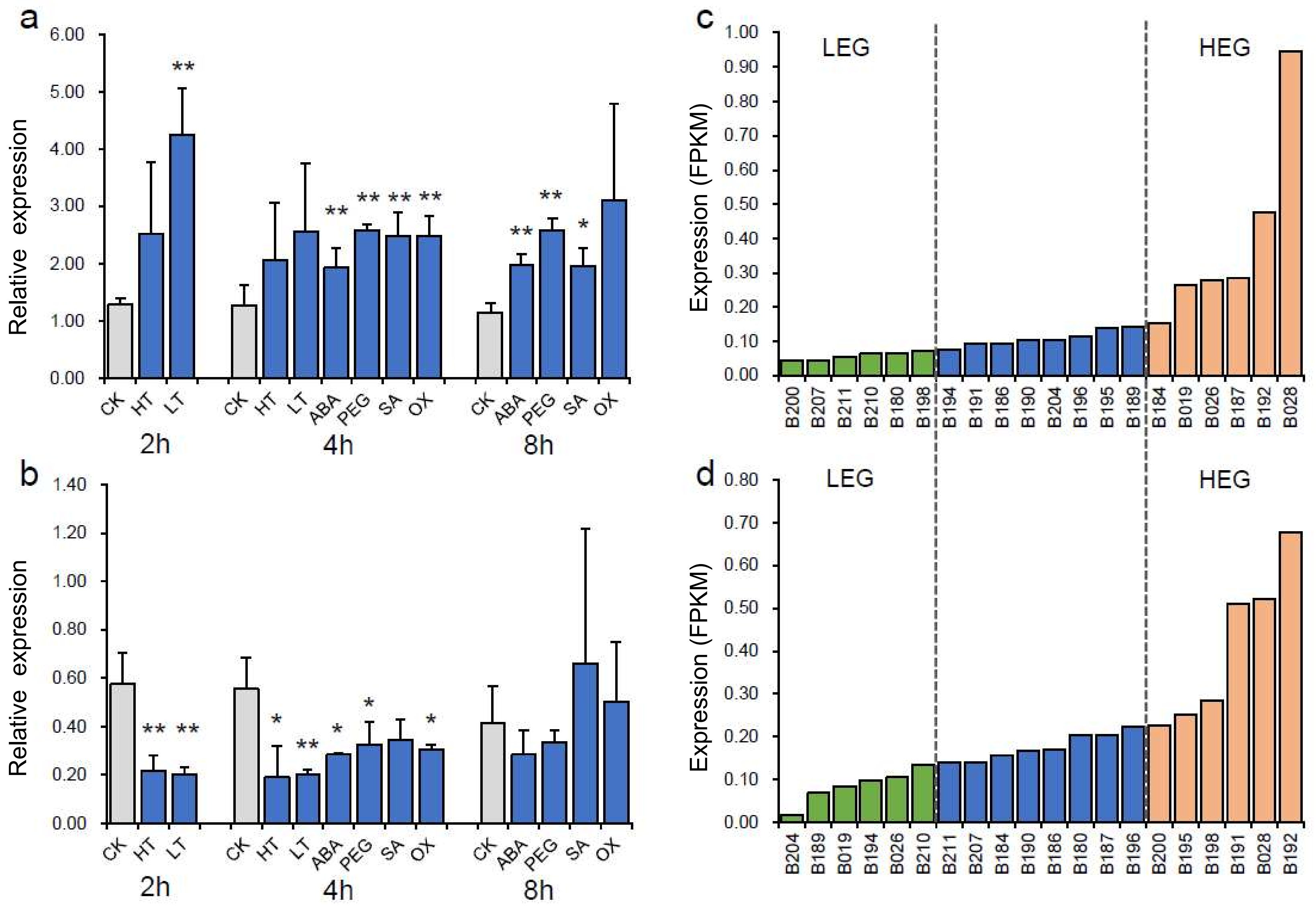

Figure S17. Expression of *orfX* and *rps4* quantified by qPCR. **a**. Expression of *orfX* under diverse treatments. **b.** Expression of *rps4* under diverse treatments. **c**. Expression of *orfX* among twenty genotypes. **d**. Expression of *rps4* among twenty genotypes. CK: normal condition; LT: low temperature; HT: high temperature; SA: salinity; OX: oxidative; LEG: low expression genotypes; HEG: high expression genotypes. * and ** indicate significant differences between CK and treatments at $p<0.05$ and $p<0.01$ by independent t test. ATP6 is used as the reference.

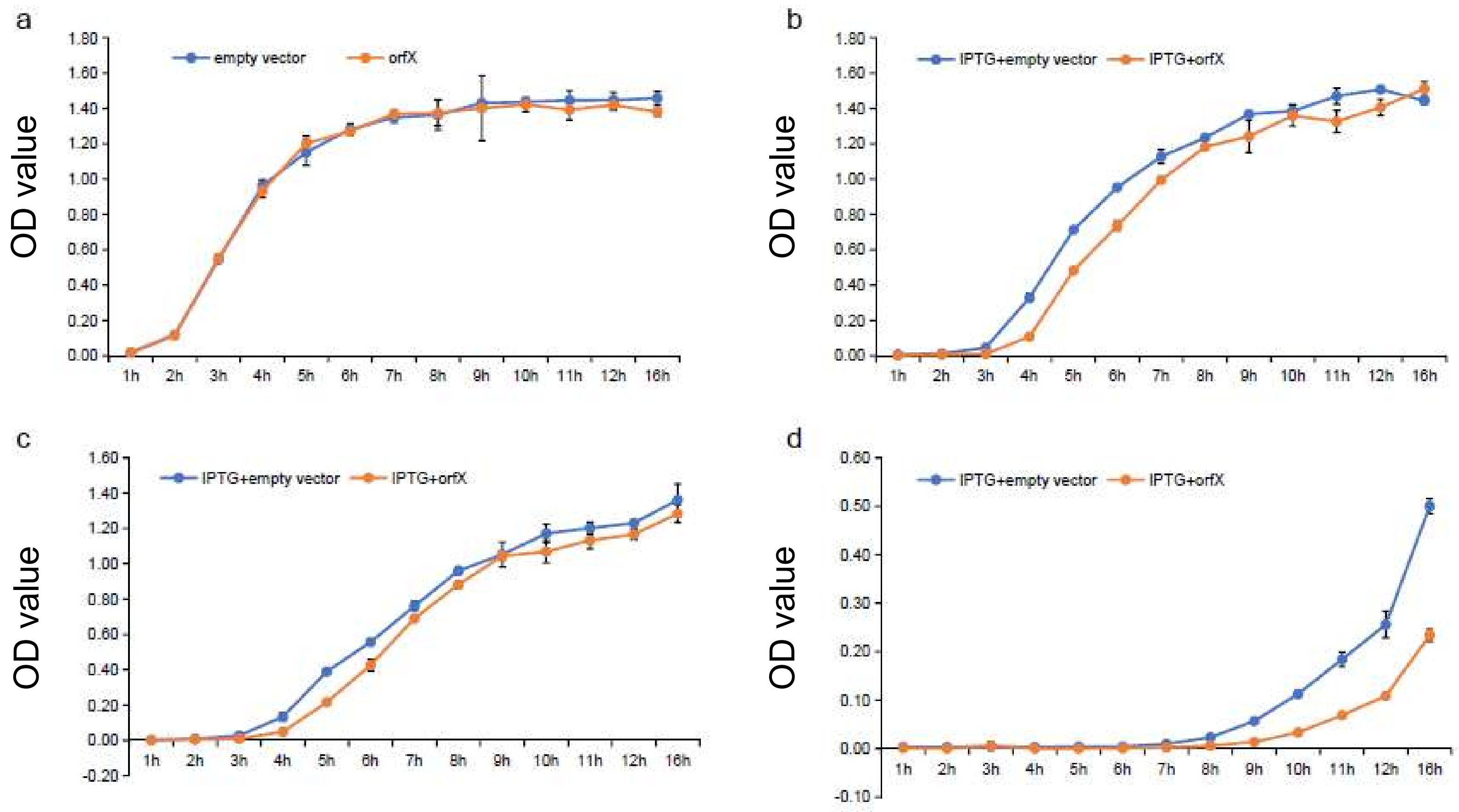

Figure S18. The growth performance of E coli transformed with pGEX-6p-1 and pGEX-6p1-orfX recombinants. Transformed E. coli were subjected to different treatments. a. LB medium only, b. LB medium with IPTG, c. LB medium with IPTG and 0.6 M sorbitol, d. LB medium with IPTG 0.5M NaCl.
